# A Robust Method for Extracting Anti-Loosening Lines for Turbine Unit Bolts Under Multi-Factor Influences

**DOI:** 10.3390/s26175411

**Published:** 2026-08-27

**Authors:** Tong Zhang, Yingbing Ran, Haipeng Gong, Tao Wu, Taide Ma, Jiang Guo, Fang Yuan

**Affiliations:** 1 Hubei Technology Innovation Center for Smart Hydropower, Wuhan 430014, China; 2025282080095@whu.edu.cn (T.Z.); gong_haipeng@ctg.com.cn (H.G.); wu_tao@cypc.com.cn (T.W.); ma_taide@cypc.com.cn (T.M.); guo.river@whu.edu.cn (J.G.); 2 School of Power and Mechanical Engineering, Wuhan University, Wuhan 430072, China; 3 China Yangtze Power Co., Ltd. (CYPC), Wuhan 430014, China

**Keywords:** bolt loosening detection, object detection, image segmentation, feature extraction, multi-head self-attention

## Abstract

Dynamic inspection of hydropower-generator rotors is hindered by long imaging distances, metallic reflections, and motion-induced degradation of anti-loosening marking lines. We present a task-decoupled vision framework that first localizes bolts and then segments their marking lines within scale-normalized regions of interest. The YOLO-SE detector combines spatial-to-depth convolution with efficient multi-scale attention to preserve small-object features and suppress illumination interference. The segmentation stage integrates a MobileViT2 backbone, attention-enhanced atrous spatial pyramid pooling, and Dice cross-entropy loss to recover global context and sparse foreground boundaries. In laboratory experiments, YOLO-SE achieved 99.4% mean average precision at 0.5 intersection-over-union (mAP50) and 99.8% recall, exceeding YOLO26n by 3.2 and 3.4 percentage points, respectively. The complete segmenter achieved 98.67% mean intersection-over-union (mIoU) and 95.38% target-line IoU; across five seed-matched runs, YOLO-SE obtained 99.32 ± 0.10% mAP50 and the segmenter obtained 98.61 ± 0.09% mIoU. Site-specific validation on M12 rotor-clamp bolts supported field applicability under changes in scale, viewpoint, and illumination. The complete cascade required 53.1 million parameters, 207.7 GFLOPs, 2.38 GB peak GPU memory, and 31.8 ms per frame, meeting a 30 FPS GPU budget under the evaluated conditions.

## 1. Introduction

Bolted connections are critical to the structural integrity of large rotating machinery, including hydropower-generator rotors. Cyclic loading, vibration, and impact can reduce bolt preload and compromise rotor balance and operational safety [1,2,3]. Manual torque inspection is labor intensive, whereas contact sensors can be difficult to deploy at scale or unsuitable for continuous remote monitoring. Machine vision therefore provides an attractive non-contact approach for bolt localization, rotation estimation, and preload-loss assessment [1,2,3].

Visual loosening assessment relies on preserving bolt geometry and anti-loosening marking lines throughout the imaging pipeline. Hydropower-turbine chambers impose three coupled challenges. Long safety clearances reduce each bolt to a small region of the panoramic image. Supplementary lighting produces non-uniform reflections on metallic surfaces, and rotor motion during exposure can blur slender marking lines. Motion blur is particularly consequential because it can remove the high-frequency evidence needed to separate the line from the metal background before network inference. Together, these factors cause spatial-information loss, background interference, and fragmented line boundaries.

Early vision-based methods relied on handcrafted features. Cha et al. combined edge and Hough-transform features with a support vector machine [4]; Ramana et al. used Viola–Jones localization followed by support-vector classification [5]; and Park et al. developed an image-based method for wind-turbine tower bolts [6]. Although interpretable, these pipelines depend on stable gradients and well-resolved geometry. Metallic reflections, non-uniform illumination, and weak edges can therefore produce false responses or remove the features required for classification.

Recent non-contact monitoring has progressed from marker localization to direct geometric and pixel-level inference. YOLOv5-based marker detection supports angle estimation across changes in distance, viewpoint, and illumination [7], while keypoint methods combined with synthetic data reduce the need for exhaustive physical sampling [8]. These developments also reflect the broader challenge of representing small objects in modern detectors [9]. Semantic segmentation with perspective correction supports arrangement-independent measurement [10], and lightweight detector–segmenter systems permit real-time identification of anti-loosening lines [11]. High-resolution cross-scale Transformers further strengthen global-local representations of bolt geometry [12].

Field-oriented systems have integrated detection, tracking, and segmentation for operating hydraulic-turbine rotors [13], and explainable visual targets have improved interpretation of the inferred loosening state [14]. However, most recent pipelines assume that sufficiently visible local evidence remains after localization. They do not directly address the coupled case in which a bolt occupies few panoramic pixels and its marking line is further weakened by reflection, low contrast, and motion-induced smear. Macro-level localization and slender-line reconstruction consequently require different representations, motivating a scale-decoupled cascade rather than direct full-frame segmentation.The results of the review are presented in Table 1.

The present study contributes a task-conditioned integration of established components rather than a new constituent module. The detector preserves weak bolt evidence and suppresses reflection-dominated responses before scale normalization, after which the segmenter reconstructs sparse marking-line boundaries within context-preserving ROIs. Unlike a generic detector–segmenter assembly, the stage interface, module placement, and evaluation protocol are designed around the macro-to-micro scale mismatch of hydropower-rotor imaging. The contribution therefore lies in the scale-specific placement of SPD-Conv, EMA, MobileViT2, CBAM, and Dice-based optimization, together with adaptive ROI transfer and matched laboratory and field evaluation.

(1) A macro-localization and micro-segmentation framework with adaptive ROI expansion separates small-object detection from slender-line reconstruction. This decomposition reduces the spatial-scale burden on a single network while preserving context around the nut–base interface.

(2) Detector and segmenter components are assigned according to their target degradation mechanisms. SPD-Conv retains spatial samples during downsampling, EMA recalibrates reflection-dominated responses, MobileViT2 models long-range context, and A-ASPP with Dice cross-entropy loss emphasizes sparse multi-scale boundaries.

(3) A dual-source evaluation combines controlled laboratory disturbances with field images from a hydropower rotor. The experiments assess scale, viewpoint, illumination, and bolt-specification changes and report the laboratory-to-field performance gap to define the current applicability boundary.

## 2. Methodology

### 2.1. Overview

**Figure 1:**
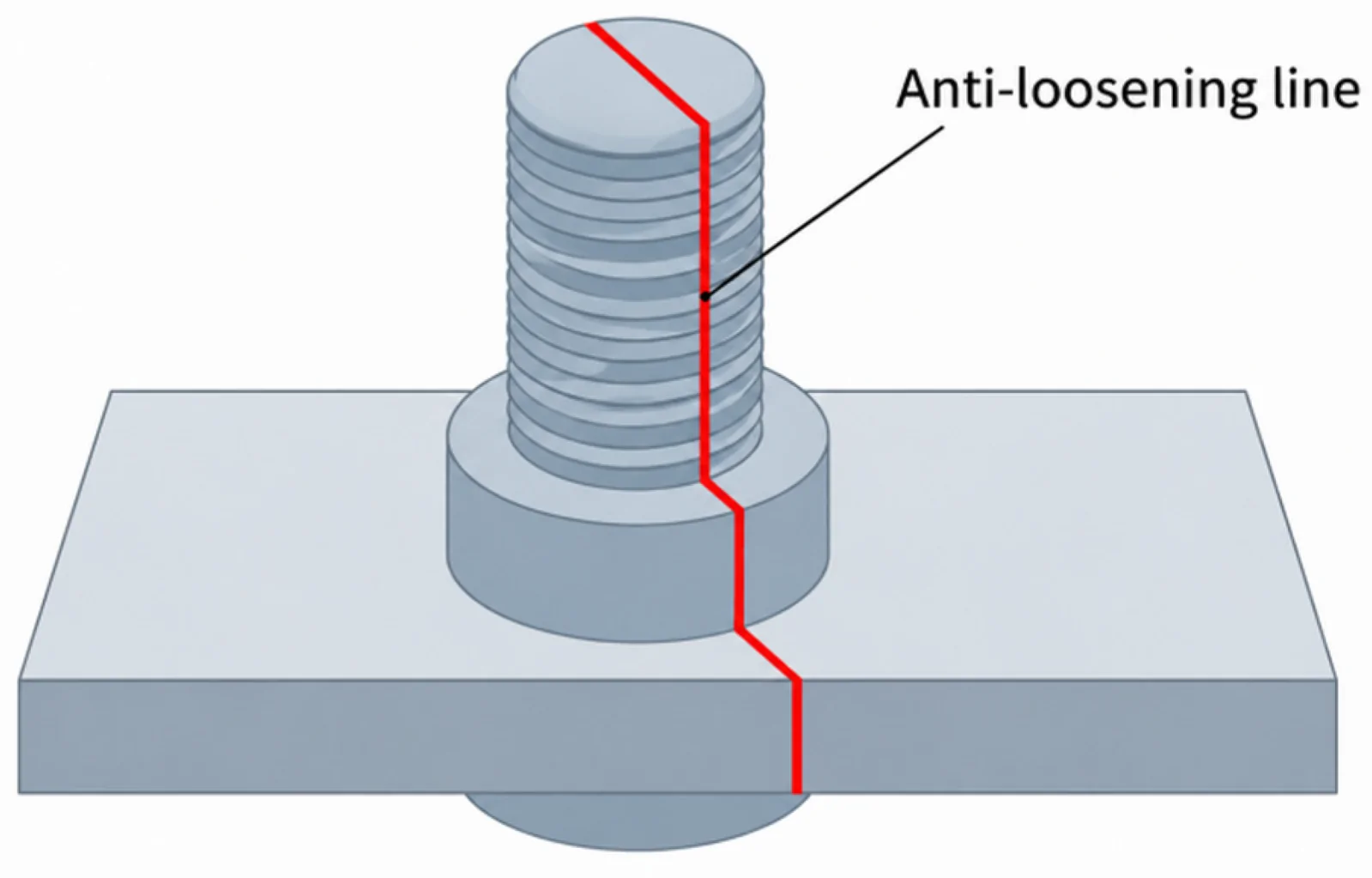
Bolt marking-line configuration.

Anti-loosening marking lines provide a direct visual reference for monitoring rotor bolts. When a bolt rotates relative to its base, the line becomes discontinuous across the nut–base interface. The displacement between the two line segments is geometrically related to the relative rotation angle, allowing non-contact visual estimation of bolt loosening.An example of a loosening prevention line is shown in Figure 1.

**Figure 2:**
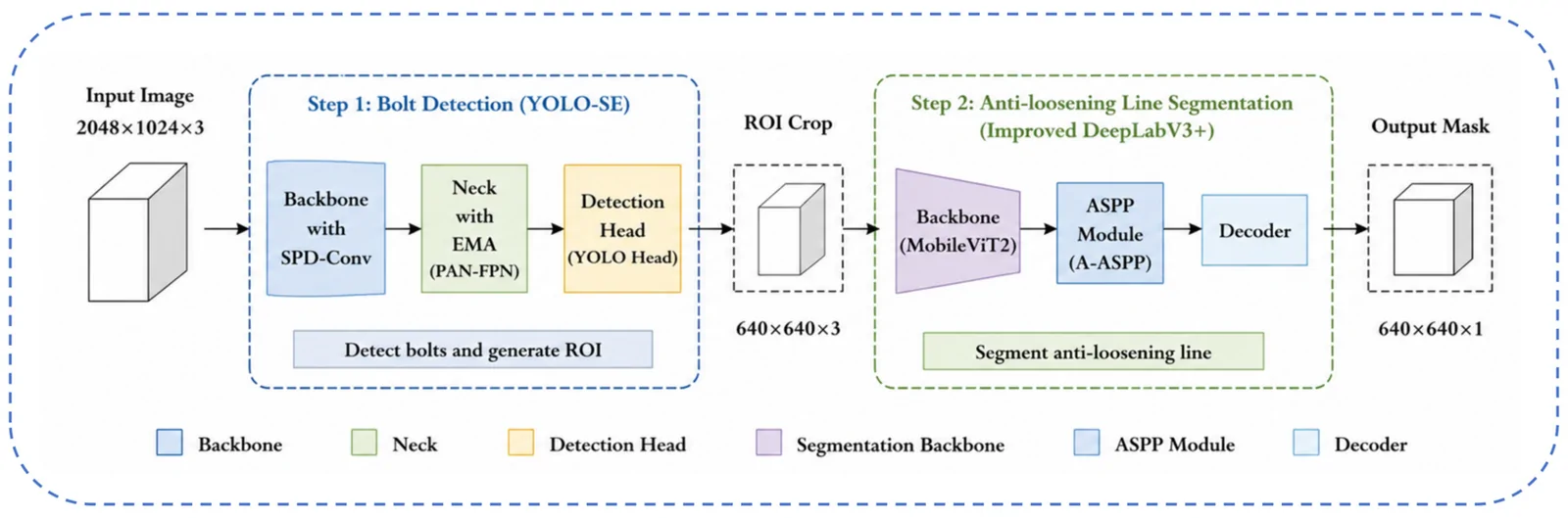
Overall architecture of the proposed framework.

The framework decouples two tasks that operate at incompatible spatial scales. Direct segmentation of a 2048 × 1024 panoramic frame allocates most pixels to background and repeatedly downsamples the sparse marking-line signal. The proposed cascade instead maximizes bolt recall at panoramic scale and normalizes each expanded ROI to 640 × 640 pixels for boundary-focused segmentation. This design preserves context around the nut–base interface while increasing the foreground-pixel proportion presented to the segmenter. Figure 2 summarizes the three processing stages.

(1) Macro-level target localization: YOLO-SE processes each high-resolution panoramic image and localizes bolt assemblies across the tested distances, viewpoints, and illumination conditions. The predicted boxes are then expanded to form context-preserving ROIs.

(2) Context-preserving ROI construction: Each predicted box is expanded around its center to retain the nut–base interface and adjacent marking-line context. The resulting ROI is resized to 640 × 640 pixels before segmentation.

(3) Micro-level semantic reconstruction: Three task-specific models segment the nut region, nut-side marking line, and base-side marking line. Long-range context modeling and boundary-aware optimization are used to recover discontinuous line evidence and produce spatially coherent masks.

### 2.2. Bolt Detection and ROI Extraction Using an Improved YOLO26

The detector stage must localize small bolt assemblies with high recall in panoramic images acquired at long distances and under variable viewpoints and illumination. Direct full-frame segmentation repeatedly downsamples these targets and can remove their geometric structure. We therefore modify the YOLO26 downsampling and feature-fusion stages and then apply adaptive ROI extraction to normalize target scale before segmentation.

#### 2.2.1. YOLO26 Network Structure

**Figure 3:**
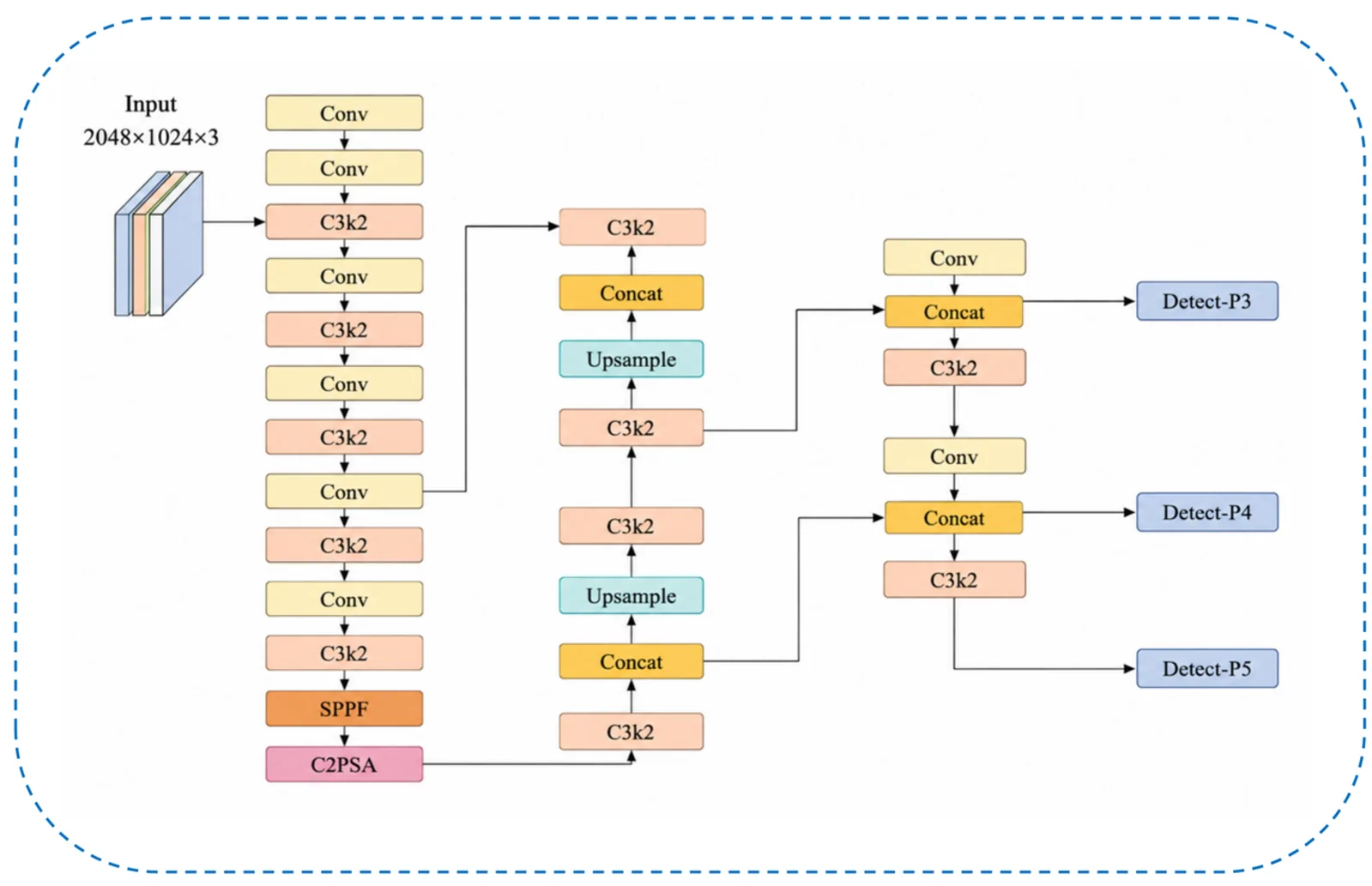
Baseline YOLO26 architecture redrawn from the official YOLO26 description [15].

YOLO26n was selected as the detector baseline because its single-stage architecture provides a practical accuracy-speed reference and exposes clearly defined downsampling and feature-fusion stages. The selection does not imply universal superiority over other detectors. Instead, YOLO26n provides a compact baseline for isolating the effects of SPD-Conv and EMA. The network architecture of YOLO26n is shown in Figure 3.

#### 2.2.2. SPD-Conv Module

The conventional YOLO26 backbone uses stride-2 convolutions for downsampling. Although these layers enlarge the receptive field, their sampling pattern can weaken spatial evidence from small targets.

**Figure 4:**
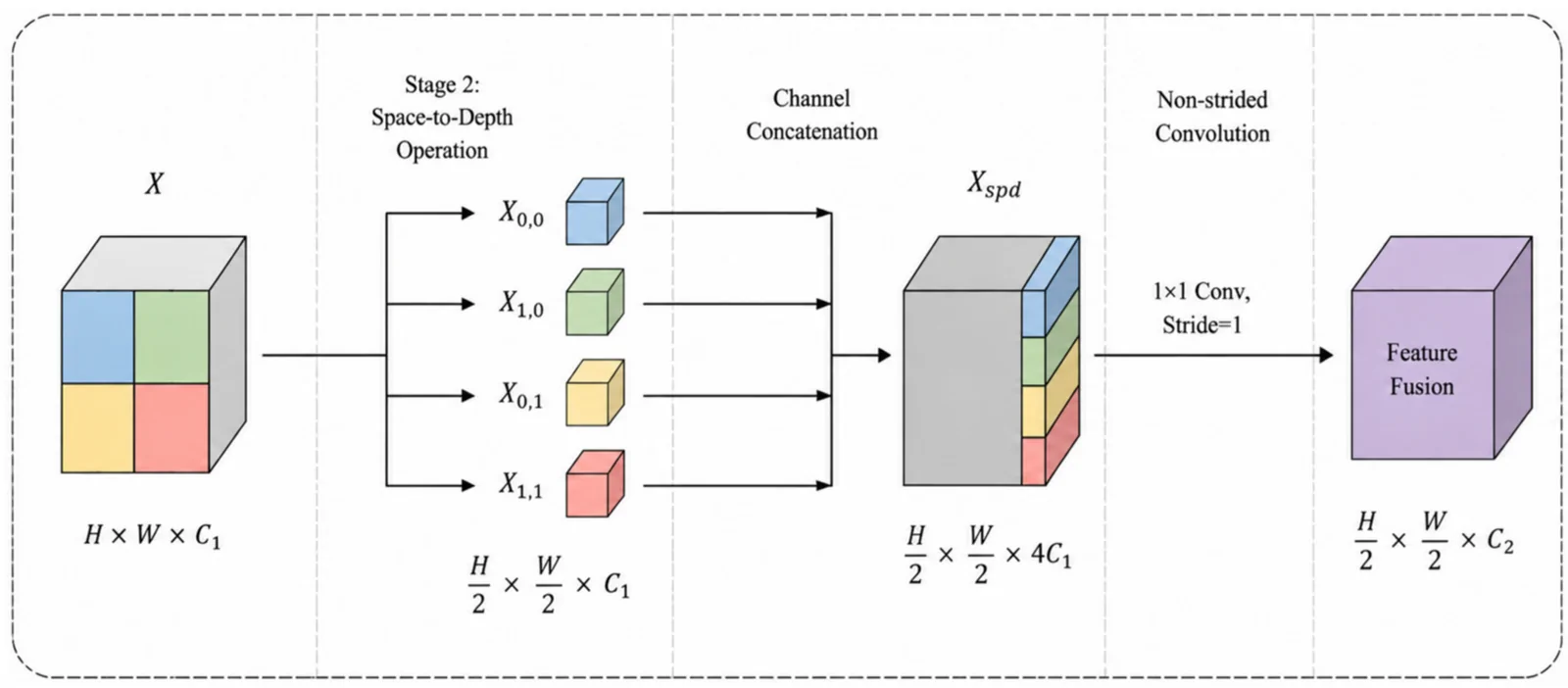
Structure of SPD-Conv, adapted from [16].

SPD-Conv therefore replaces the stride-2 convolutions at the P3/8, P4/16, and P5/32 backbone stages. It rearranges spatial samples into the channel dimension before applying a non-strided convolution, avoiding direct deletion of sampled pixels [16].Its network architecture is shown in Figure 4.

Let the input backbone feature map $XS\times S\times C_{1}X_{0,0},X_{1,0},X_{0,1},X_{1,1}\frac{S}{2}\times \frac{S}{2}\times C_{1}$ at a given stage have dimensions. The SPD module performs periodic subsampling of the feature map with a step size of 2, dividing it into four spatially complementary sub-feature map blocks, each with half the original resolution. Its mathematical slicing mapping process can be formally expressed as:

(1) $$X_{0,0}=X\left[0:S:2,0:S:2,:\right],\;X_{1,0}=X[1:S:2,0:S:2,:]$$

(2) $$X_{0,1}=X\left[0:S:2,1:S:2,:\right],\;X_{1,1}=X[1:S:2,1:S:2,:]$$

The four submaps are concatenated along the channel dimension to form $\frac{S}{2}\times \frac{S}{2}4C_{1}X_{spd}$. This rearrangement halves each spatial dimension and increases the channel dimension fourfold.

(3) $$X_{spd}=Concat\left(X_{0,0},X_{1,0},X_{0,1},X_{1,1}\right)$$

A non-strided convolution produces $3\times 3\frac{S}{2}\times \frac{S}{2}\times C_{2}$, adjusts the output channel dimension, and introduces nonlinear channel interactions. Because spatial reduction is achieved by rearrangement rather than sample deletion, fine-scale evidence remains available in the channel representation.

#### 2.2.3. EMA Mechanism

**Figure 5:**
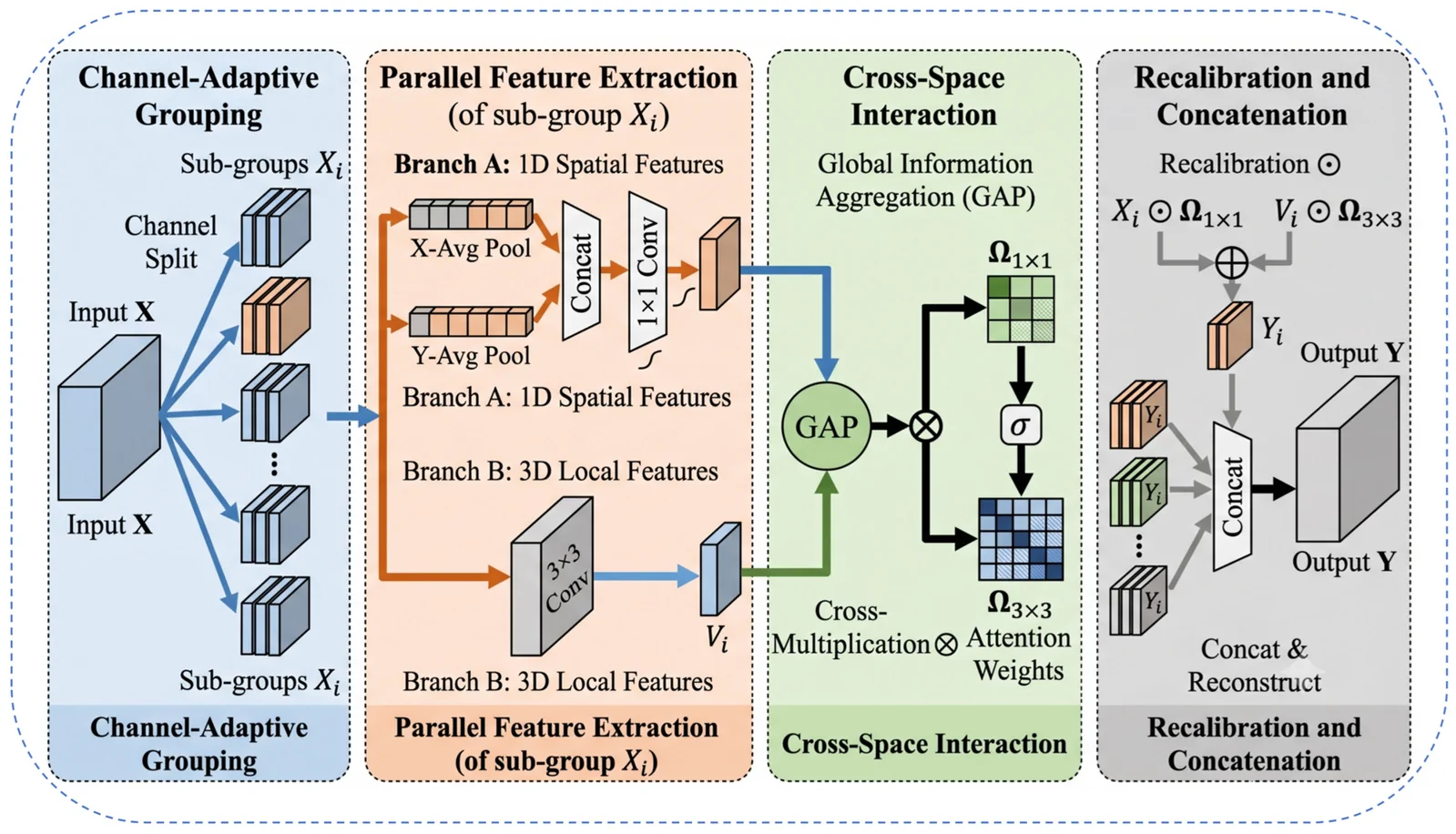
Principle of the EMA module, redrawn from [17].

Dynamic industrial lighting introduces strong reflection-dominated responses into the feature space. An efficient multi-scale attention (EMA) module is inserted after the YOLO26 feature-fusion block to reduce their amplification. EMA retains channel-specific information and uses cross-spatial interactions to reweight informative bolt features [17].The EMA network architecture is shown in Figure 5.

For an input feature map $X\in R^{C\times H\times W}XGX_{i}\in R^{(C/G)\times H\times W}$, EMA partitions the channels into G subgroups to reduce computation while retaining diverse semantic cues. Each subgroup is processed by two parallel branches.

The first branch applies one-dimensional adaptive average pooling along the horizontal and vertical axes. For channel c, the aggregated descriptors $z_{c}H$ and $z_{c}W$ encode positional context along height h and width w, respectively.

(4) $$z_{c}^{H}\left(h\right)=\frac{1}{W}\sum_{j=1}^{W}X_{i}\left(c,h,j\right),\;z_{c}^{W}(w)=\frac{1}{H}\sum_{k=1}^{H}X_{i}(c,k,w)$$

The horizontal and vertical descriptors are concatenated and passed through a shared $1\times 1F_{1\times 1}T^{H}T^{W}$ transformation. The activated output is then split along the spatial dimension to generate two coordinate-attention vectors.

The second branch maps each subgroup feature through $3\times 3X_{i}V_{i}$ to obtain local multi-scale context.

Cross-branch interaction is implemented through $GAPX_{i}V_{i}⊗$, which combines global pooling and matrix multiplication to aggregate subgroup and local multi-scale features into a joint attention map.

(5) $$\Omega_{1\times 1}=\sigma \left(F_{1\times 1}\left(T^{H}\right)⊗GAP(V_{i})\right)$$

(6) $$\Omega_{3\times 3}=\sigma \left(F_{1\times 1}\left(T^{W}\right)⊗GAP(X_{i})\right)$$

The σ⊙ operators apply sigmoid normalization and element-wise multiplication. The resulting weights recalibrate the corresponding feature maps before fusion.

(7) $$Y_{i}=X_{i}⊙\Omega_{1\times 1}+V_{i}⊙\Omega_{3\times 3}$$

The recalibrated subgroup outputs $Y_{i}$ are concatenated to restore the original feature dimensions. This operation increases the relative response of bolt regions while suppressing illumination-dominated background features.

#### 2.2.4. Bolt ROI Extraction

For each input frame, YOLO-SE predicts the candidate bolt assemblies. Each detection is represented by a five-dimensional vector containing the bounding-box geometry and classification confidence:

(8) $$B_{t,i}=[x_{c},y_{c},w,h,p{]}^{T}$$

Here, $\left(x_{c},y_{c}\right)$ denotes the absolute coordinates of the target bounding box’s center point in the pixel coordinate system; w and h represent the absolute width and height of the bounding box, respectively; and p∈(0,1,1) denotes the classification confidence score assigned by the network, used to indicate whether a target bolt exists within that region.

Each detected bolt is cropped from the original image and passed to the segmentation stage. Because the marking line can extend from the nut surface onto the surrounding base, cropping at the predicted box boundary can truncate the target line.

We therefore expand each predicted box about its center to 1.5 times its original width and height. The expanded ROI boundaries are computed from the original box parameters and clipped to the physical image limits:

(9) $$\left\{\begin{array}{c} w_{new}=\;1.5\cdot w\\ h_{new}=\;1.5\cdot h\\ x_{min}=\max\left(0,x_{c}-\frac{w_{new}}{2}\right)\\ y_{min}=\max\left(0,y_{c}-\frac{h_{new}}{2}\right)\\ x_{max}=\min\left(W_{img},x_{c}+\frac{w_{new}}{2}\right)\\ y_{max}=\min\left(H_{img},x_{c}+\frac{h_{new}}{2}\right) \end{array}\right.$$

The expanded ROI contains the complete nut body, the adjacent base surface, and the visible continuation of the marking line.

### 2.3. Improving DeepLabV3+ for Three-Stage Segmentation

**Figure 6:**
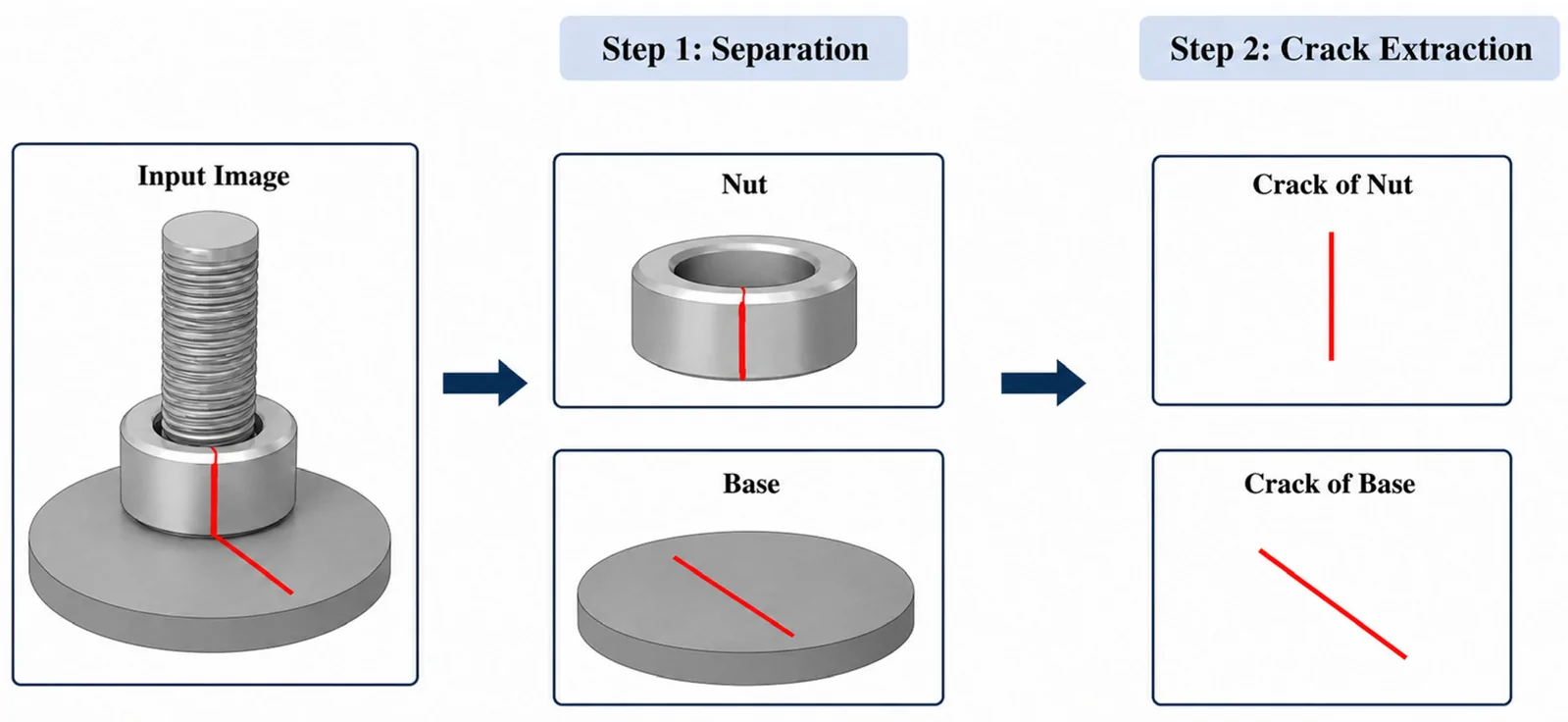
Three-stage segmentation process for the nut region, nut-side marking line, and base-side marking line.

After ROI extraction, three task-specific models segment the nut region, nut-side marking line, and base-side marking line. Figure 6 illustrates this micro-level processing sequence.

#### 2.3.1. Mixed CNN-Transformer Backbone Network

**Figure 7:**
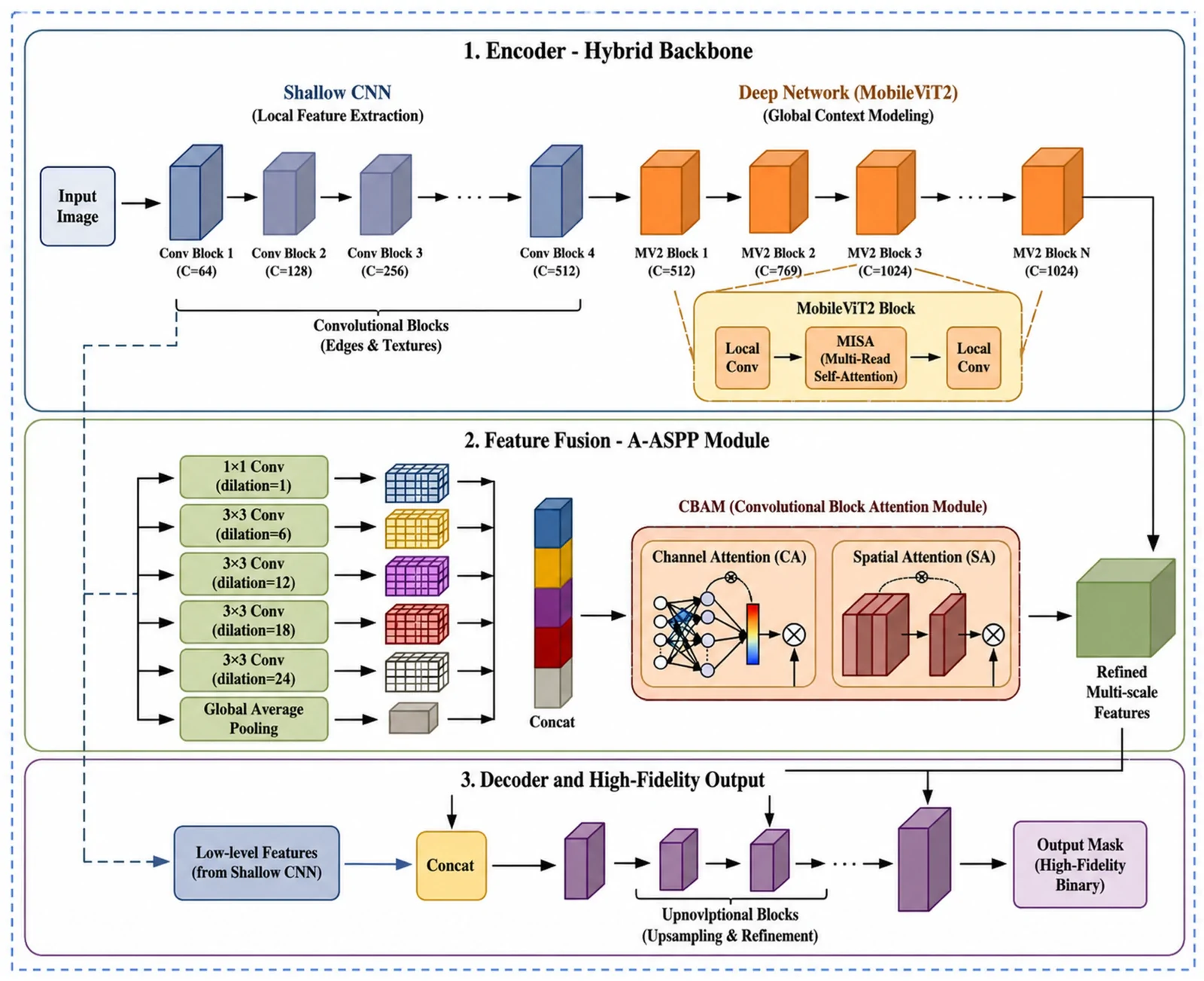
Improved DeepLabV3+ architecture assembled by the authors; the MobileViT2 and CBAM blocks follow [19,20].

DeepLabV3+ [18] serves as the segmentation baseline. Its encoder and multi-scale fusion stages are modified to improve reconstruction when line contrast is weak or spatially discontinuous (Figure 7).

The ResNet-50 encoder is replaced by a MobileViT2 hybrid backbone that combines convolutional local representations with lightweight Transformer-based context modeling.

Shallow convolutional layers retain local edges and textures, whereas deeper MobileViT2 blocks partition the feature tensor into patches and model long-range dependencies through separable self-attention [19].

(10) $$Attention(Q,K,V)=Softmax(\frac{QK^{T}}{\sqrt{d_{k}}})V$$

Here, Q, K, and V denote the query, key, and value projections of the input features, respectively; the scaling term is defined in the equation above.

#### 2.3.2. Attention-Enhanced Multi-Scale Pooling

A convolutional block attention module (CBAM) [20] is added after the original ASPP fusion stage to form A-ASPP. CBAM sequentially recalibrates the fused feature map through channel and spatial attention to reduce reflection and background responses.

The channel-attention submodule aggregates spatial information by global average and maximum pooling. A multilayer perceptron then estimates an importance weight for each channel.

(11) $$M_{c}(F)=\sigma (MLP(AvgPool(F))+MLP(MaxPool(F)))$$

Channel attention consequently reduces the contribution of feature channels dominated by background noise.

The spatial-attention submodule compresses the recalibrated feature map along the channel dimension. A large-kernel convolution then produces a spatial weight map that emphasizes high-response image regions.

(12) $$M_{s}(F^{\prime })=\sigma (f^{7\times 7}([AvgPool(F^{\prime });MaxPool(F^{\prime })]))$$

Element-wise multiplication applies the learned weights to the feature map, increasing the relative response of slender marking-line pixels.

#### 2.3.3. Boundary-Aware Joint Loss Function

Anti-loosening marking lines can occupy less than 1% of an ROI, creating severe foreground–background imbalance. Under cross-entropy alone, background pixels can dominate the optimization gradient, a problem related to the dense-detection imbalance addressed by focal loss [21].

We therefore combine cross-entropy with Dice loss, which directly optimizes foreground overlap and is well suited to sparse segmentation targets [22].

(13) $$L_{total}=\lambda_{1}L_{CE}+\lambda_{2}L_{Dice}$$

The pixelwise cross-entropy loss is defined as:

(14) $$L_{CE}=-\frac{1}{N}\sum_{i=1}^{N}\sum_{c=1}^{C}g_{i,c}\log(p_{i,c})$$

The Dice loss is defined as:

(15) $$L_{Dice}=1-\frac{2\sum_{i=1}^{N}p_{i,1}g_{i,1}+\epsilon }{\sum_{i=1}^{N}p_{i,1}+\sum_{i=1}^{N}g_{i,1}+\epsilon }$$

In these equations, N is the total number of pixels, yi is the one-hot ground-truth label for pixel i, pi is the predicted class probability, and ε prevents division by zero. The Dice term directly optimizes foreground overlap, thereby increasing the contribution of the sparse target class and promoting continuous boundaries. Cross-entropy and Dice loss were assigned equal weights of 1.0 throughout segmentation training.

## 3. Experiments and Field Validation

### 3.1. Laboratory Experiments and Dataset Construction

**Figure 8:**
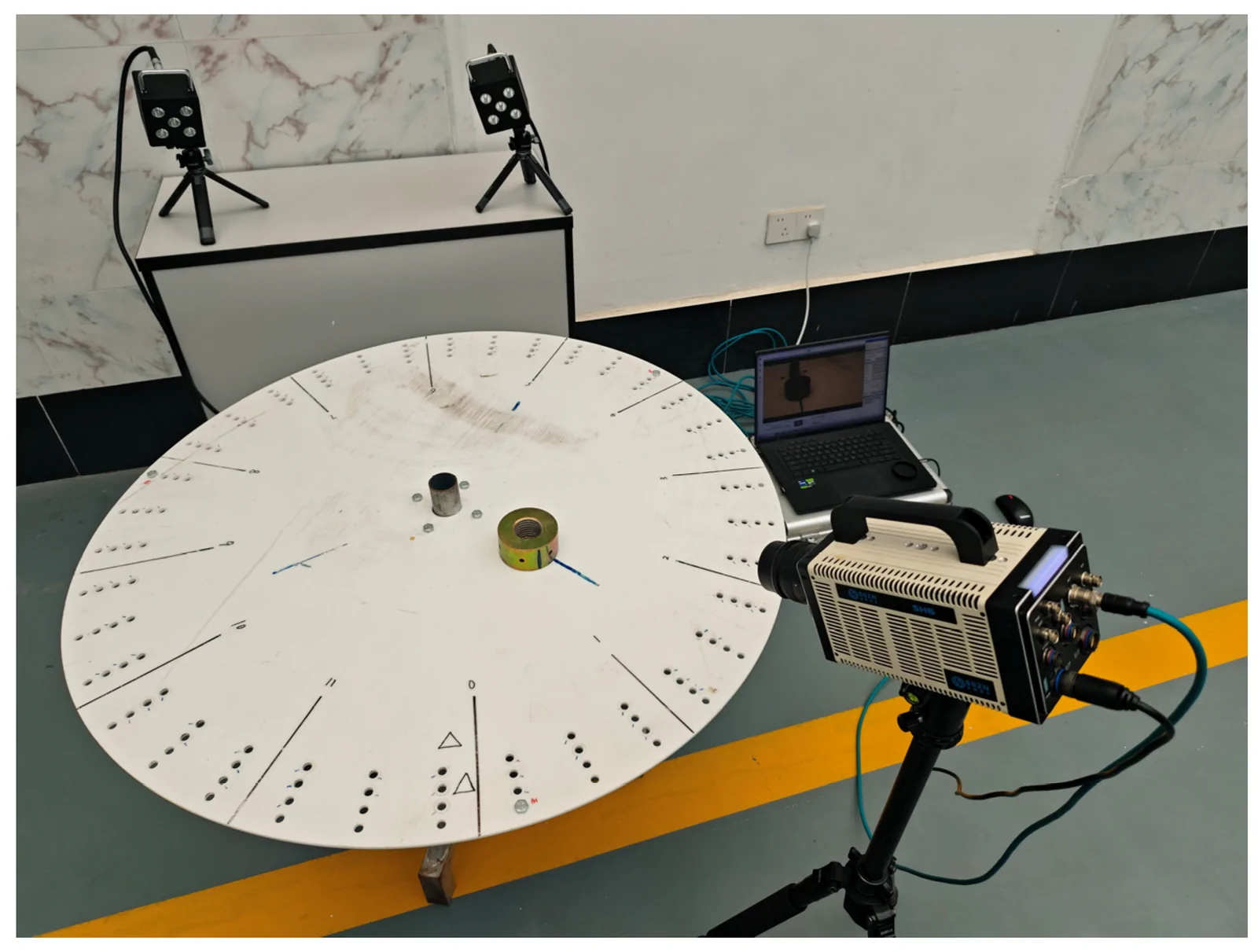
Schematic of the laboratory test bench.

The evaluation used a controlled laboratory dataset and an independently acquired field dataset. Laboratory acquisition varied imaging distance, illumination, camera height, and viewpoint on the dynamic test bench shown in Figure 8. The field dataset was collected at the Unit 10F rotor-maintenance site and included smaller M12 bolts, oblique views, non-uniform illumination, and partial occlusion by oil contamination. This design supports controlled robustness analysis and site-specific assessment of field applicability.

Laboratory images were acquired on a customized dynamic rotation test bench using ten M42 bolt assemblies matching the specification used at a domestic hydropower station. Anti-loosening marking lines were applied to the nut and base surfaces with industrial markers following field practice. A DeepView industrial camera recorded images at up to 2048 × 1024 pixels.

**Figure 9:**
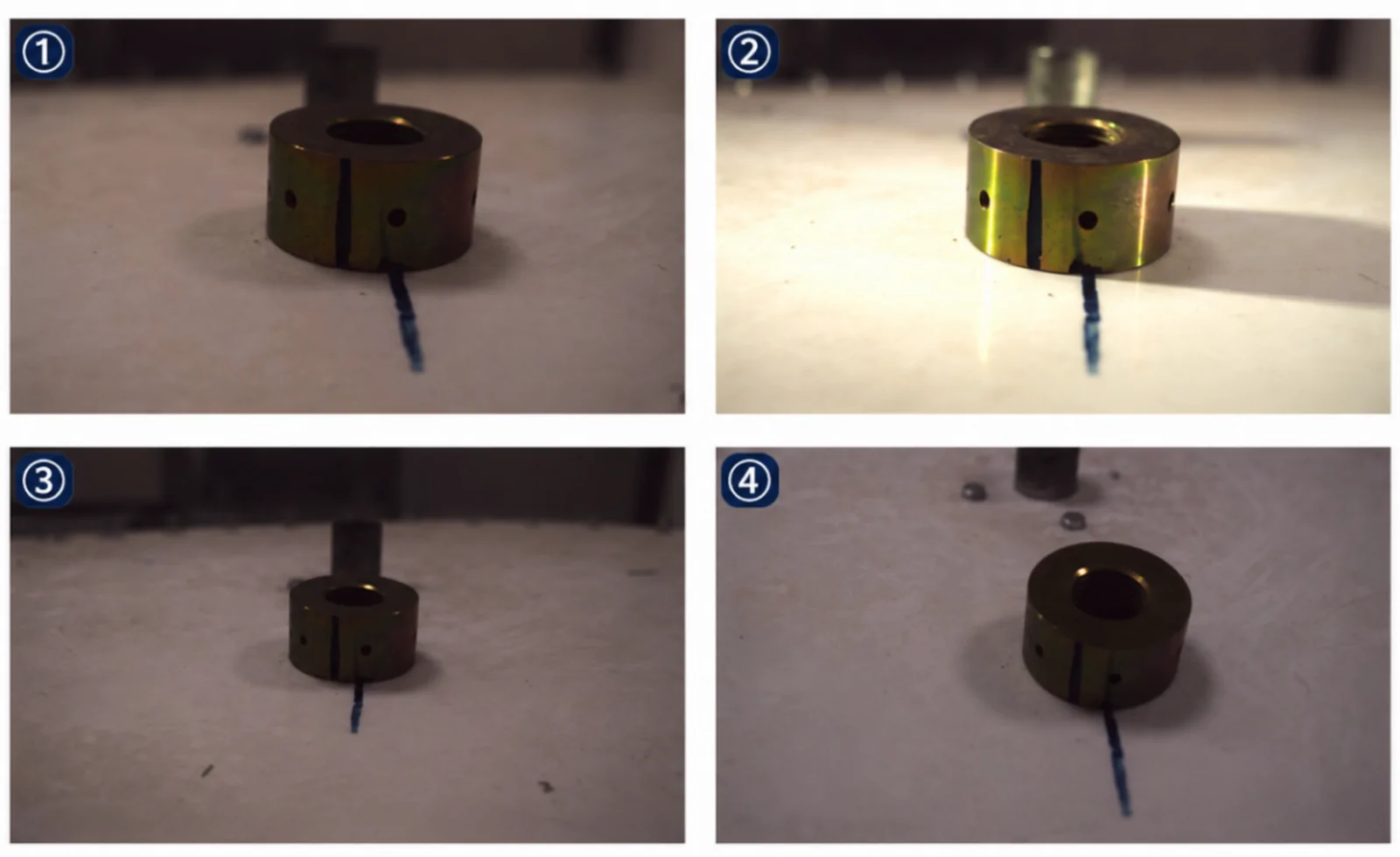
Representative bolts under different laboratory imaging conditions. Figures ①–④ show images of the nut under various shooting conditions; Figures ①, ②, and ③ depict images taken at θ = 20° under different lighting and distance conditions, respectively; Figure ④ shows the image taken at θ = 40°.

Laboratory acquisition varied two groups of physical and imaging factors:

(1) Viewpoint and scale: The top-view angle ranged from 10° to 50°, the imaging distance from 0.2 to 0.7 m, and the camera height from 0.1 to 0.6 m.

(2) Illumination and acquisition coverage: Illumination was varied from 0% to 40% of the experimental lighting range. The protocol comprised 190 acquisition groups with 30 sampled images per group, yielding 5700 laboratory images (Table 2). Some example figures are shown in Figure 9. Motion blur occurred in practical videos but was not independently controlled through rotor speed, exposure time, blur direction, or blur length. Incidental blurred frames therefore cannot support a calibrated robustness curve, and retrospective isotropic blur would not reproduce direction-dependent smear and specular integration. Motion blur is consequently excluded from the validated robustness factors.

The ten M42 assemblies were recorded across 190 source sequences at 30 frames s^−1^. Bounding boxes and three segmentation targets were annotated in LabelMe: the nut region, nut-side marking line, and base-side marking line. Boxes enclosed the visible bolt assembly, and polygons followed visible foreground boundaries at full resolution. Occluded pixels were labeled only when the class boundary remained distinguishable, while uncertain regions were resolved by consensus. All 5700 images were annotated. Independent reannotation of a random 10% subset yielded a mean bounding-box IoU of 0.96 and a mean mask Dice score of 0.95. To limit leakage between adjacent frames, complete source sequences were assigned to the training and held-out partitions in an 8:2 ratio.

### 3.2. Model Training and Ablation Experiments

Training followed two stages. YOLO-SE was first optimized for bolt detection, after which three DeepLabV3+ models were trained for the nut region, nut-side marking line, and base-side marking line. Table 3 summarizes the hardware and software environment.

Unless otherwise stated, the principal models used random seed 0, while the repeated-run analysis used seeds 0-4. The detector was initialized from official COCO-pretrained YOLO26n weights and trained with mosaic augmentation, HSV color jitter, horizontal flipping, translation, and scale perturbation [23]. After a three-epoch linear warm-up, the learning rate decayed from 0.001 according to a cosine schedule. Segmentation backbones were initialized from ImageNet-pretrained weights and used a polynomial schedule with power 0.9. Checkpoints were selected by validation mAP50 for detection and validation mIoU for segmentation. On the RTX 4090 platform, detector training required approximately 5.3 GPU-hours, and each segmentation model required approximately 4.2 GPU-hours. All compared methods used the same sequence-level partition, preprocessing, and evaluation code. Method-specific hyperparameters were selected during development before final held-out evaluation.

Detection performance was measured using precision, recall, and mean average precision at an IoU threshold of 0.5 (mAP50) [24]. Segmentation performance was assessed using overall accuracy (OAcc), mean intersection-over-union (mIoU), target-line IoU (TL-IoU), and boundary F1 score (BF1).

These metrics are defined from the numbers of true positives (TP), false positives (FP), true negatives (TN), and false negatives (FN).

Precision and recall are defined as:

(16) $$P=\frac{TP}{TP+FP}$$

(17) $$R=\frac{TP}{TP+FN}$$

Overall accuracy is defined as:

(18) $$OAcc=\frac{TP+TN}{TP+TN+FP+FN}$$

Intersection-over-union (IoU) and mean IoU are defined as:

(19) $$IoU=\frac{TP}{TP+FP+FN}$$

(20) $$mIoU=\frac{1}{C}\sum_{i=1}^{C}{IoU}_{i}$$

Here, K is the number of classes. For marking-line segmentation, TL-IoU is the single-class IoU of the marking-line foreground and directly measures recovery of the sparse target.

#### 3.2.1. YOLO-SE Training and Ablation

**Figure 10:**
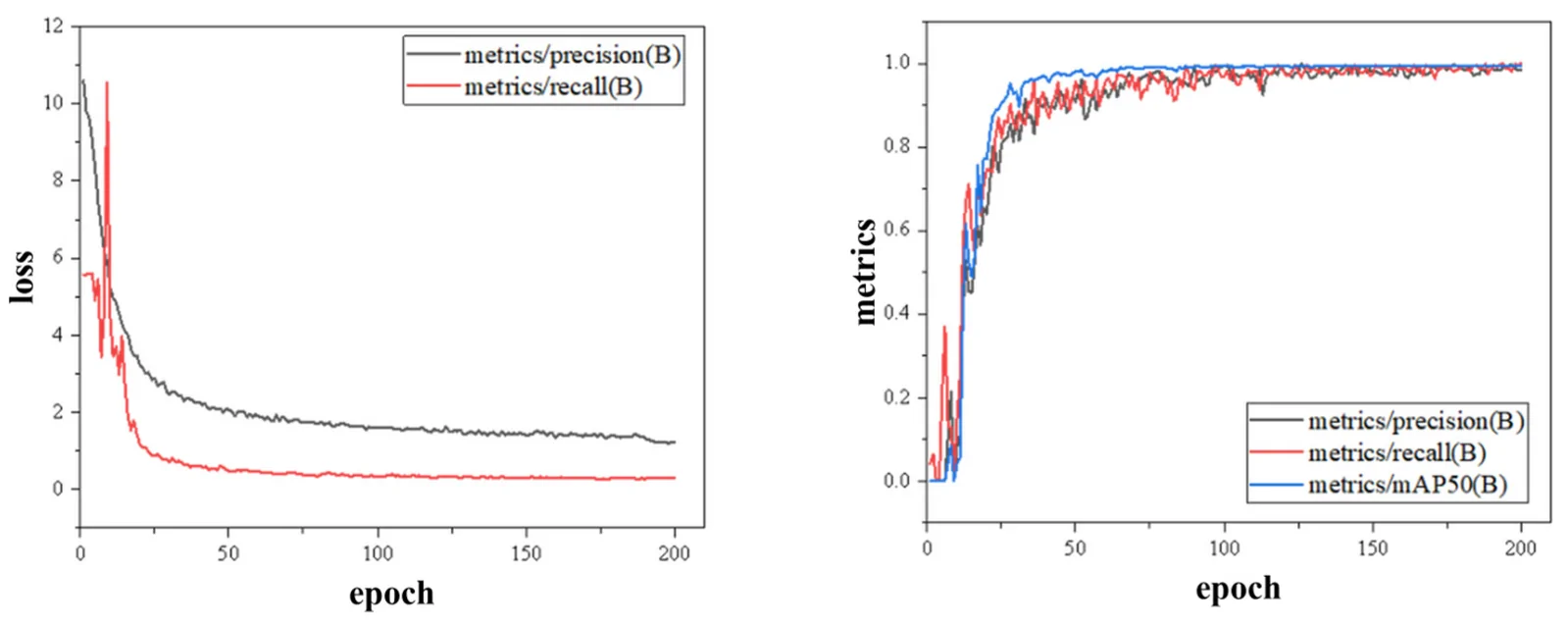
YOLO-SE training curves.

The detector used the sequence-level 8:2 partition described above. COCO-pretrained YOLO26n weights were fine-tuned for 300 epochs with AdamW, a batch size of 16, and an initial learning rate of 0.001 [25]. Consistent with the official YOLO26 formulation [15], the objective contains classification and bounding-box regression terms but does not use Distribution Focal Loss. Figure 10 shows the training curves.

(21) $$Loss=Loss_{box}+Loss_{cls}+Loss_{dfl}$$

Stepwise ablations used native YOLO26n as the baseline to isolate the effects of SPD-Conv and EMA (Table 4).

The ablation results separated the contributions of SPD-Conv and EMA. Relative to YOLO26n, SPD-Conv increased precision from 94.2% to 96.8% and recall from 96.4% to 98.7%. EMA alone increased recall to 97.8% and mAP50 to 99.2%. Combining both modules produced the highest precision (97.7%), recall (99.8%), and mAP50 (99.4%). 

YOLO-SE was also compared with representative two-stage, one-stage, and Transformer-based detectors, including Faster R-CNN, YOLOv8n, YOLO11n, and RT-DETR (Table 5).

All detectors were retrained with the same sequence-level partition, 2048 × 1024 input resolution, augmentation policy, and 300-epoch budget. Official or author-maintained implementations were used. Model-specific learning rates and weight decay were selected during development without reference to held-out results.

**Figure 11:**
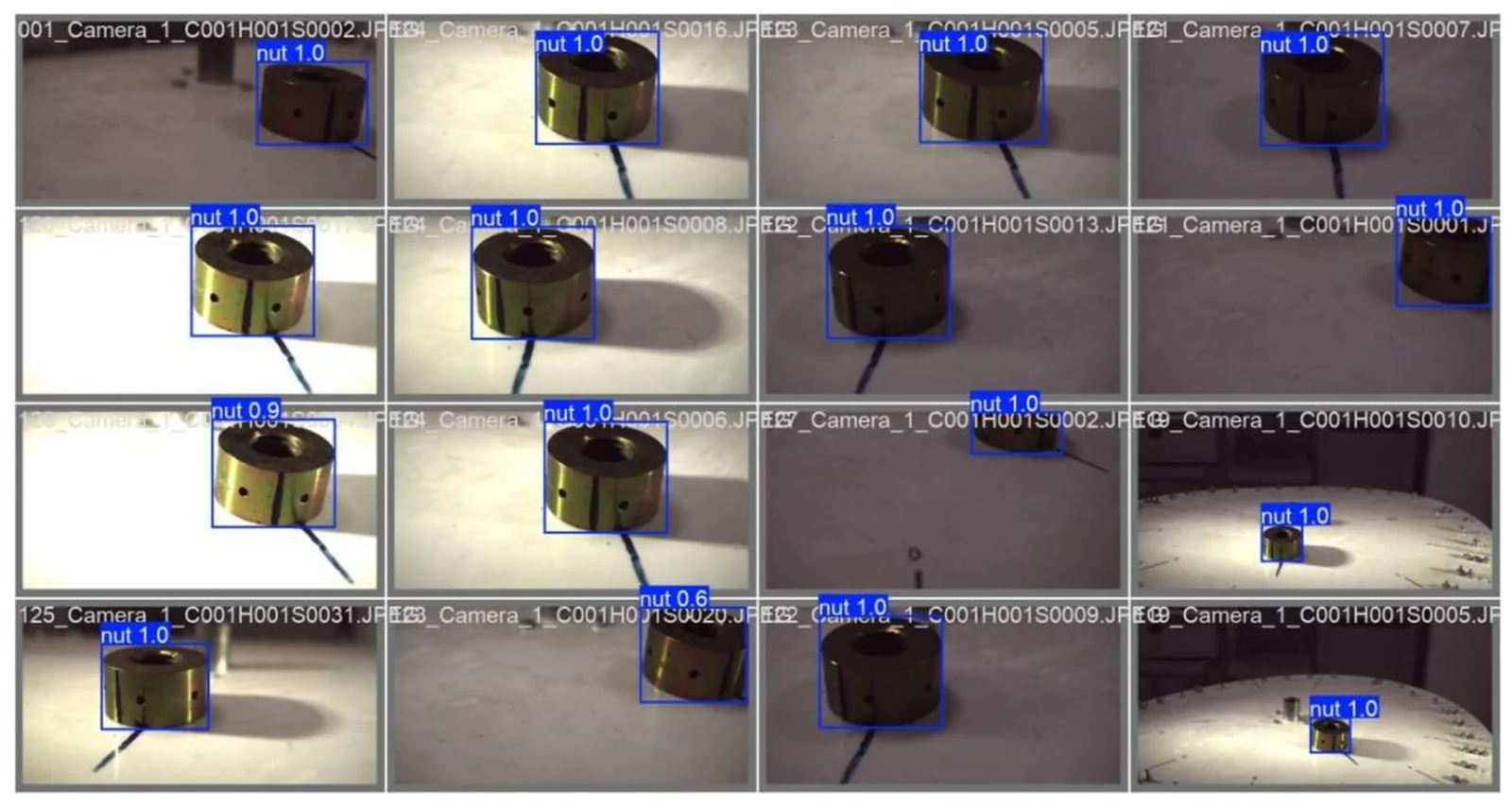
Representative YOLO-SE detections.

On the held-out partition, YOLO-SE achieved 97.7% precision, 99.8% recall, and 99.4% mAP50. GPU inference required 4.27 ms per frame (234.2 FPS), exceeding a 30 FPS real-time budget on the reported platform. Figure 11 shows representative detections.

#### 3.2.2. DeepLabV3+ Training and Segmentation Ablation

After ROI extraction, the segmentation stage was trained on 5573 cropped ROIs using the same sequence-level 8:2 partitioning principle. All three models used Adam, an initial learning rate of 0.001, polynomial decay, a batch size of 16, and 30,000 iterations.

Nut-side and base-side marking lines were annotated separately to form two additional target sets with the same partition. Their models used identical optimization settings.

The primary ablation compared the MobileViT2 backbone, Dice cross-entropy loss, and A-ASPP using mIoU and TL-IoU. Three configurations were evaluated:

Baseline: Native DeepLabV3+ with a ResNet-50 backbone, standard ASPP, and cross-entropy loss.

Model A: A MobileViT2 backbone with native ASPP and Dice cross-entropy loss.

Complete model: Model A with A-ASPP.

**Figure 12:**
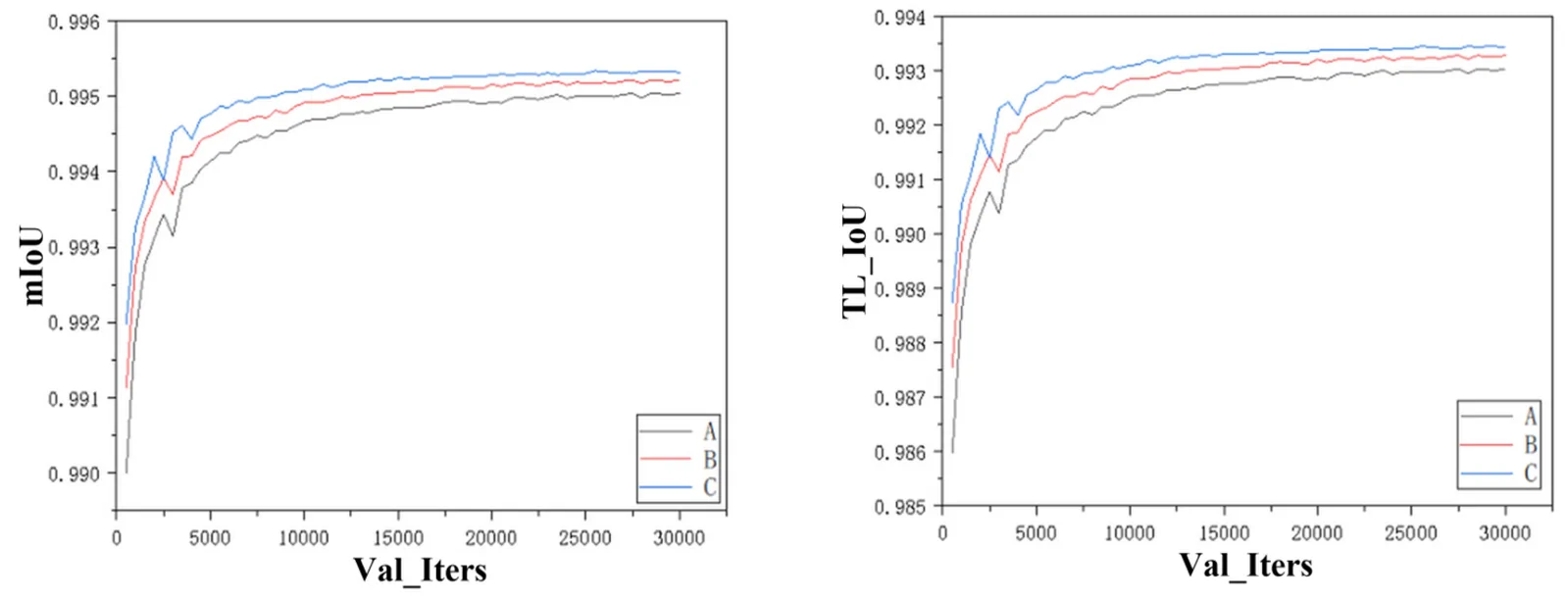
Training results for the nut-region segmentation stage.

Table 6 summarizes the best validation performance after 30,000 iterations, and Figure 12 shows the corresponding training curves. In the legend, A denotes the baseline, B denotes Model A, and C denotes the complete model.

**Figure 13:**
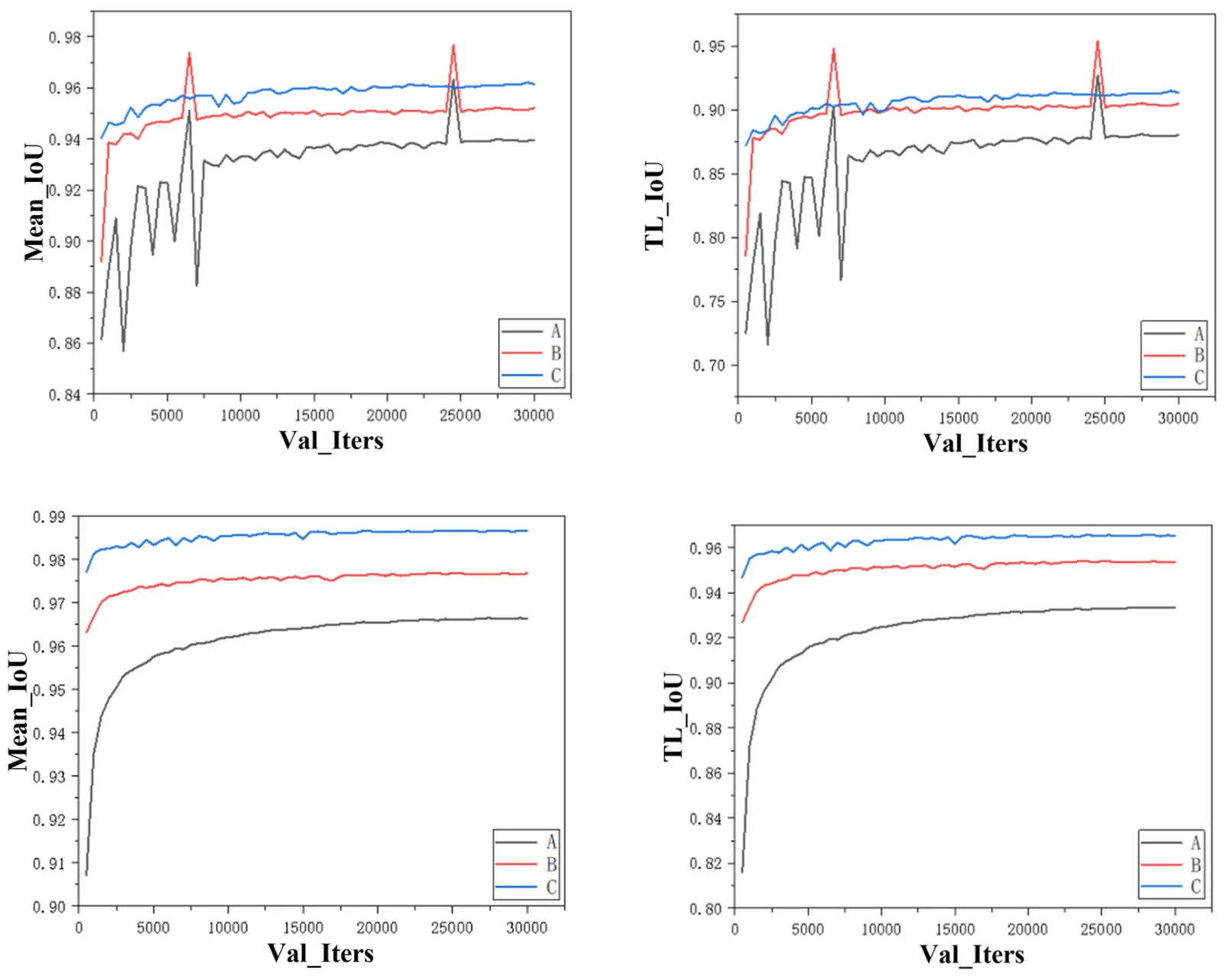
Training curves for the two marking-line segmentation stages.

Figure 13 shows the training curves for the nut-side and base-side marking-line models.

**Figure 14:**
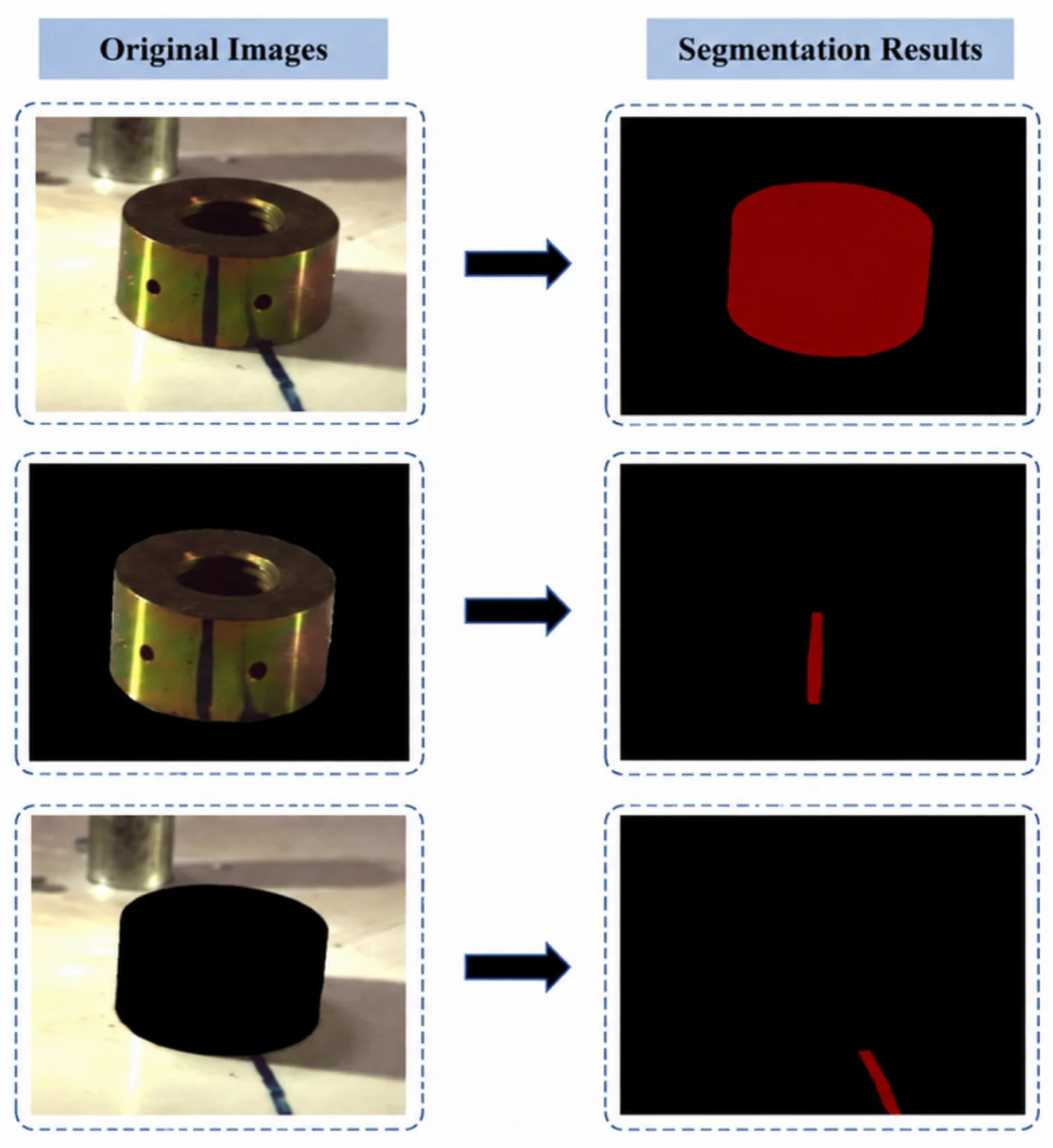
Representative segmentation results.

Representative masks are shown in Figure 14. Baseline errors were most apparent as fragmented base-side marking lines in challenging samples.

Native DeepLabV3+ achieved 96.65% mIoU and 93.36% TL-IoU. Replacing the backbone with MobileViT2 while introducing Dice cross-entropy loss increased these metrics to 97.69% and 95.42%, respectively. Adding A-ASPP further increased mIoU to 98.67%, whereas TL-IoU changed from 95.42% to 95.38%. The primary ablation therefore supports improved overall overlap but does not isolate the individual effects of MobileViT2 and Dice loss. 

Computational cost is an important deployment criterion in addition to predictive accuracy. Table 7 reports parameter count, floating-point operations, peak GPU memory, and CPU/GPU latency at batch size 1 under a unified profiling protocol.

Profiling used the hardware in Table 3, batch size 1, 50 warm-up iterations, and 1000 synchronized timed iterations. Relative to YOLO26n, YOLO-SE increased parameter count by 73.1% and FLOPs by 40.7% while improving mAP50 by 3.2 percentage points. The complete cascade required 207.7 GFLOPs and 2.38 GB peak GPU memory. GPU latency was 31.8 ms per frame (31.4 FPS), compared with 492.3 ms per frame on the CPU. The cascade therefore met a 30 FPS budget on the reported GPU but not under CPU-only inference.

Because Model A changed both the backbone and loss function, the primary ablation could not isolate their effects. Each Table 8 configuration was therefore trained independently with the same partition, optimization schedule, and checkpoint criterion. The factorial design separates MobileViT2, Dice cross-entropy loss, and A-ASPP and evaluates their pairwise and combined contributions.

Relative to native DeepLabV3+, MobileViT2 alone increased mIoU and TL-IoU by 0.45 and 0.84 percentage points, whereas Dice cross-entropy loss alone increased them by 0.69 and 1.47 points. Adding A-ASPP to Model A increased mIoU by 0.98 points but reduced TL-IoU by 0.04 points. The complete model achieved 98.67% mIoU and 95.38% TL-IoU. Dice cross-entropy loss was therefore the largest independent contributor to target-line recovery, while A-ASPP primarily improved overall region overlap.

Optimization variability was assessed using five seed-matched runs for the principal detector and segmentation configurations (Table 9). The analysis reports means, standard deviations, 95% confidence intervals, paired effect estimates, and multiplicity-adjusted comparisons.

Each configuration was trained with seeds 0–4. Values are reported as mean ± standard deviation, and 95% confidence intervals were calculated from the two-sided t distribution. Relative to its baseline, YOLO-SE improved mAP50 by 3.20 percentage points (95% CI for the paired effect, 2.89–3.51; paired two-sided t-test, Holm-adjusted *p* < 0.001). The complete segmenter improved mIoU by 2.01 points (95% CI, 1.76–2.26) and TL-IoU by 2.05 points (95% CI, 1.61–2.49), with Holm-adjusted *p* < 0.001 for both comparisons. The seed-matched independent training run was the analysis unit.

### 3.3. Robustness Evaluation

#### 3.3.1. YOLO-SE Robustness Testing

Detection robustness was assessed using the proportion of frames in which a bolt box was generated and the maximum confidence score across the controlled imaging factors.

The robustness set contained 3927 samples spanning imaging distance, illumination, camera height, and viewpoint.

(22) $$DR=\frac{N_{detected}}{N_{total}}\times 100\%$$

(23) $$CS=max\left({Conf}_{i}\right),\;i\in [1,N_{box}]$$

NDetected is the number of samples with a generated bolt box, and Ntotal is the total number of evaluated samples. The detection rate DR summarizes missed detections, while CS is the maximum predicted confidence.

**Figure 15:**
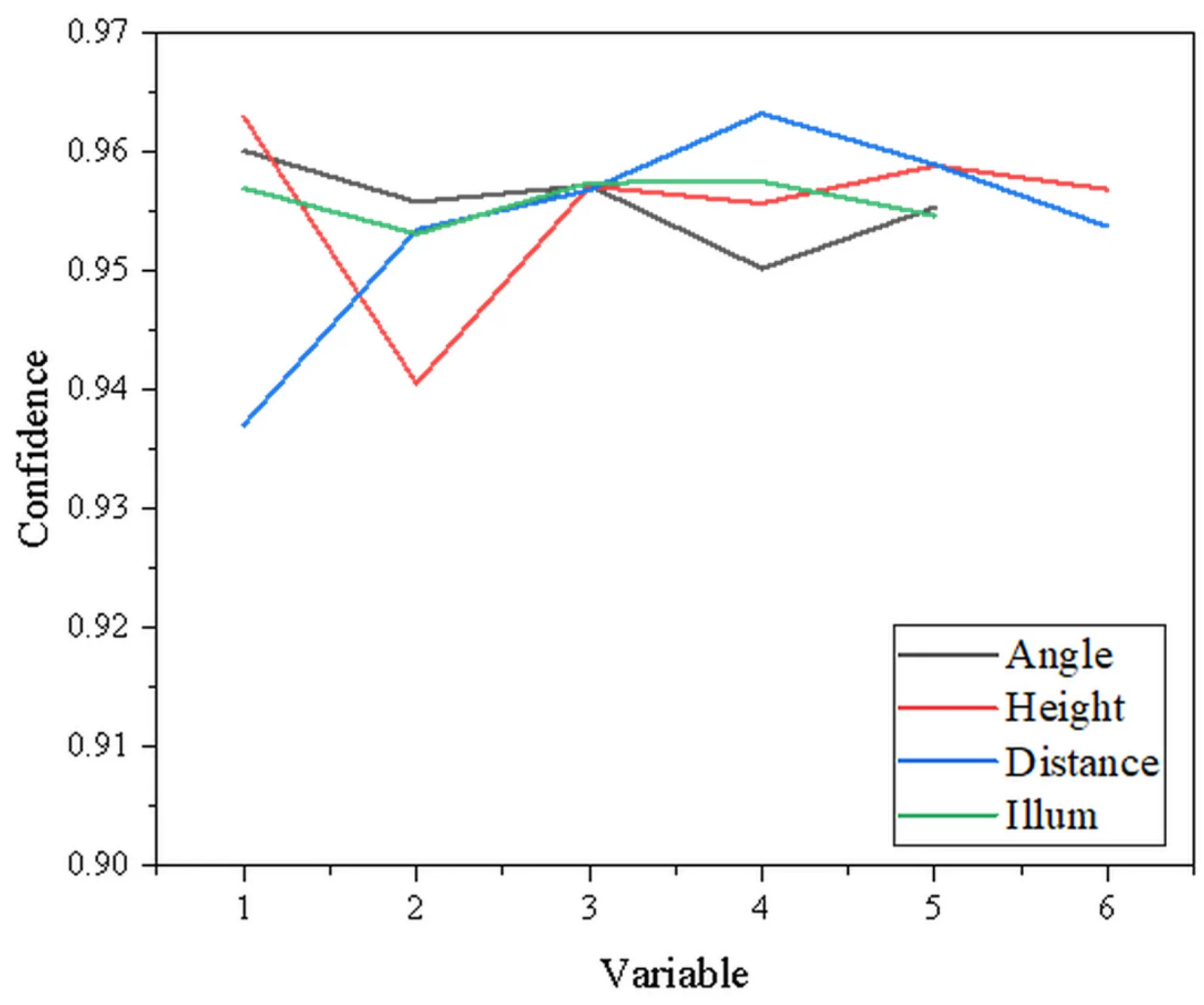
YOLO-SE confidence across the controlled imaging factors.

Figure 15 summarizes YOLO-SE confidence across the tested imaging distances, illumination levels, camera heights, and pitch angles.

Confidence remained high as imaging distance increased and the apparent bolt size decreased. It also remained stable across the tested illumination, camera-height, and viewpoint ranges. Table 10 shows that 3923 of 3927 images were detected, corresponding to a 99.898% detection rate.

#### 3.3.2. Segmentation Robustness Testing

After macro-level localization and ROI extraction, the principal segmentation challenges were boundary degradation under oblique views and local overexposure or shadow on metallic surfaces.

**Figure 16:**
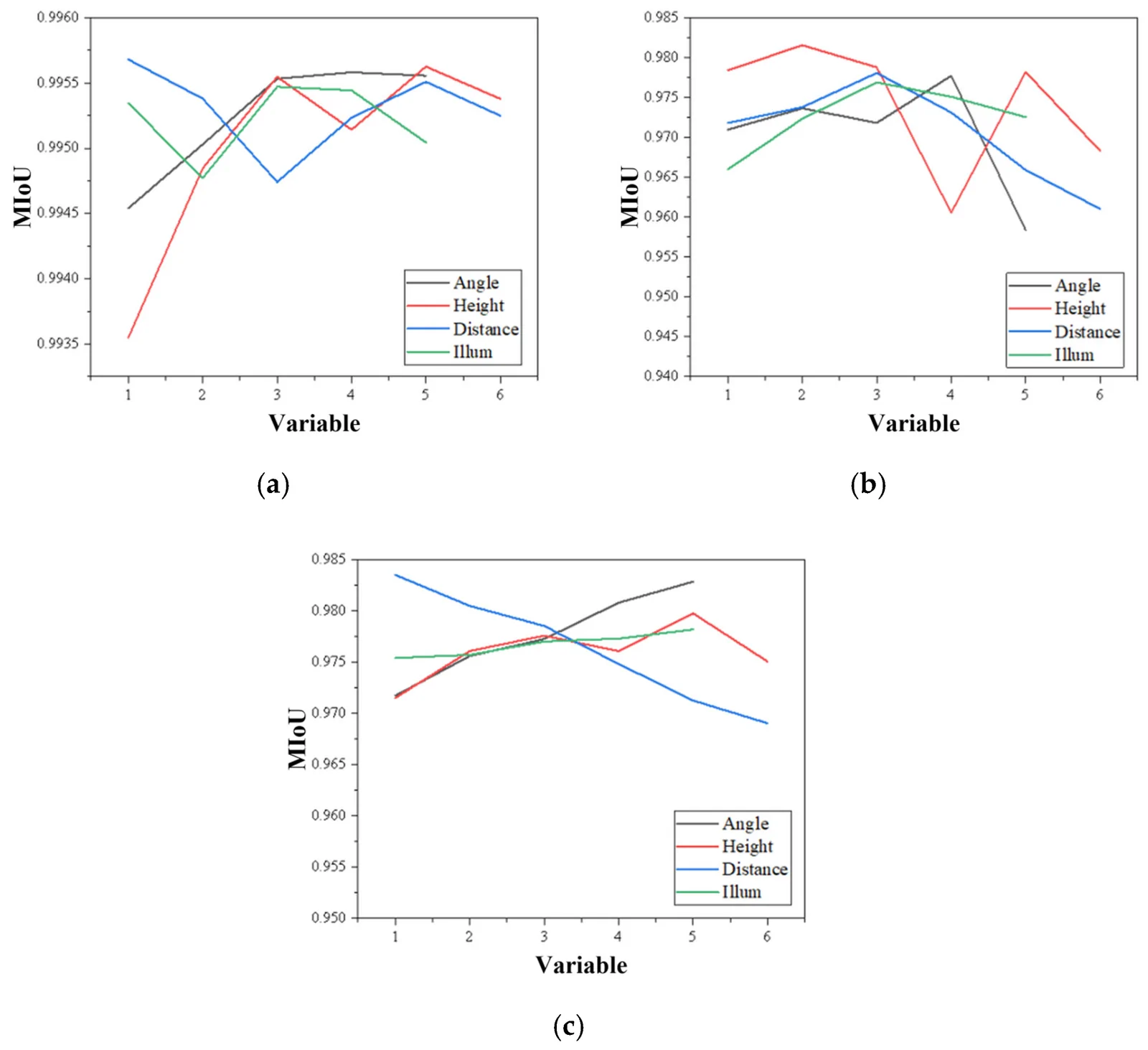
Segmentation results under the controlled imaging factors. (**a**) Nut-region segmentation result. (**b**) Nut-side marking-line segmentation result. (**c**) Base-side marking-line segmentation result.

The three segmentation models were evaluated on 5573 cropped ROIs spanning the controlled viewpoint, distance, height, and illumination conditions. Mean intersection-over-union (mIoU) and boundary F1 score (BF1) provided complementary measures of region overlap and boundary fidelity. Figure 16 shows representative results.

Across the tested viewpoints, camera heights, distances, and illumination levels, the nut-region model achieved an mIoU of 0.995. The nut-side and base-side marking-line models achieved mIoU values of 0.973 and 0.976, respectively. Their BF1 scores are reported in Table 11 and indicate high boundary fidelity within the controlled laboratory ranges.

### 3.4. Field Validation

**Figure 17:**
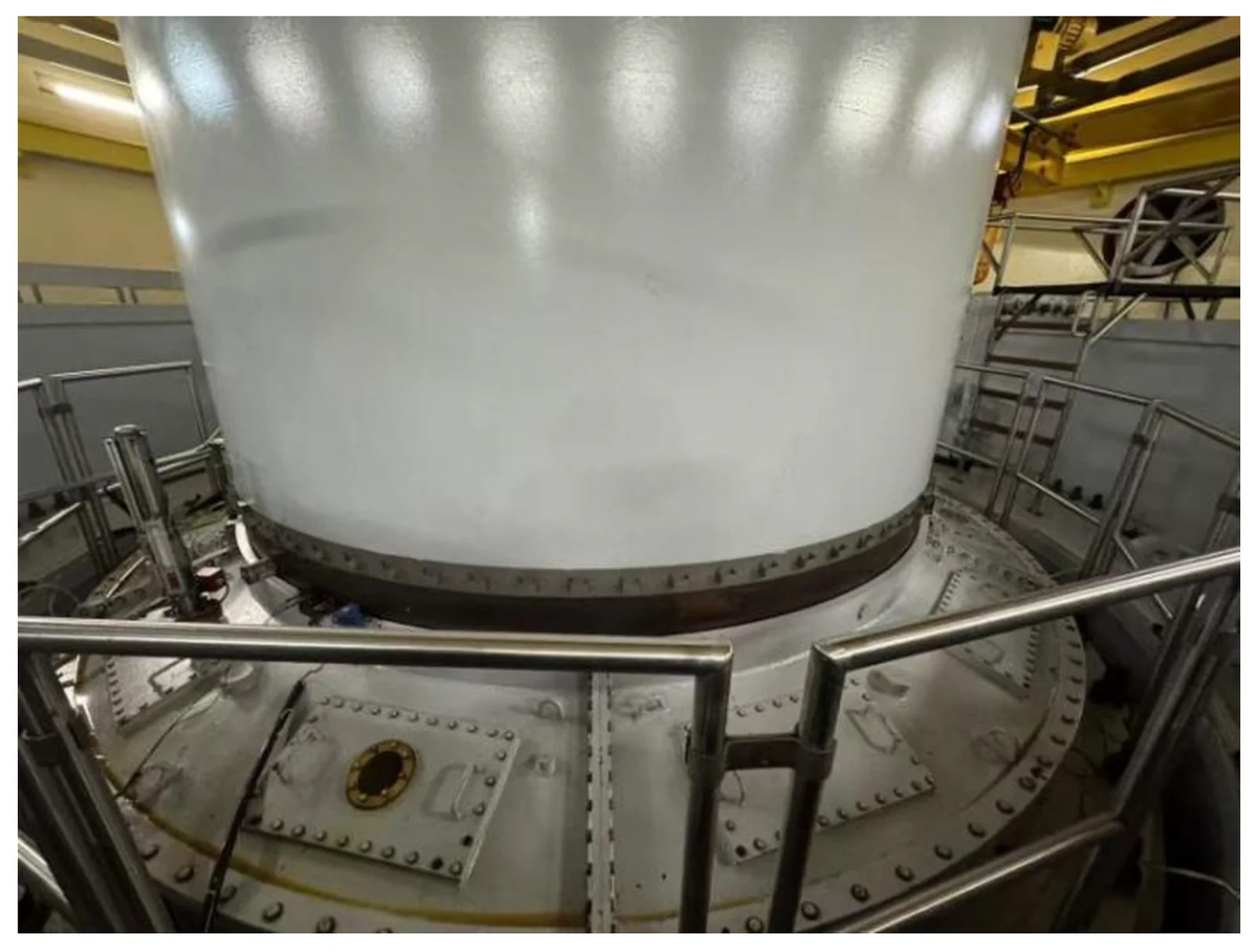
Field environment in the hydropower turbine room.

Laboratory experiments established performance under controlled conditions. Field validation was then conducted at the rotor-maintenance site of a hydropower generating unit to assess performance in the target industrial setting. Cameras were positioned approximately 120 cm from the main shaft in the Unit 10F turbine room. Figure 17 shows the test site where the target objects were bolts securing the turbine rotor clamps.

**Figure 18:**
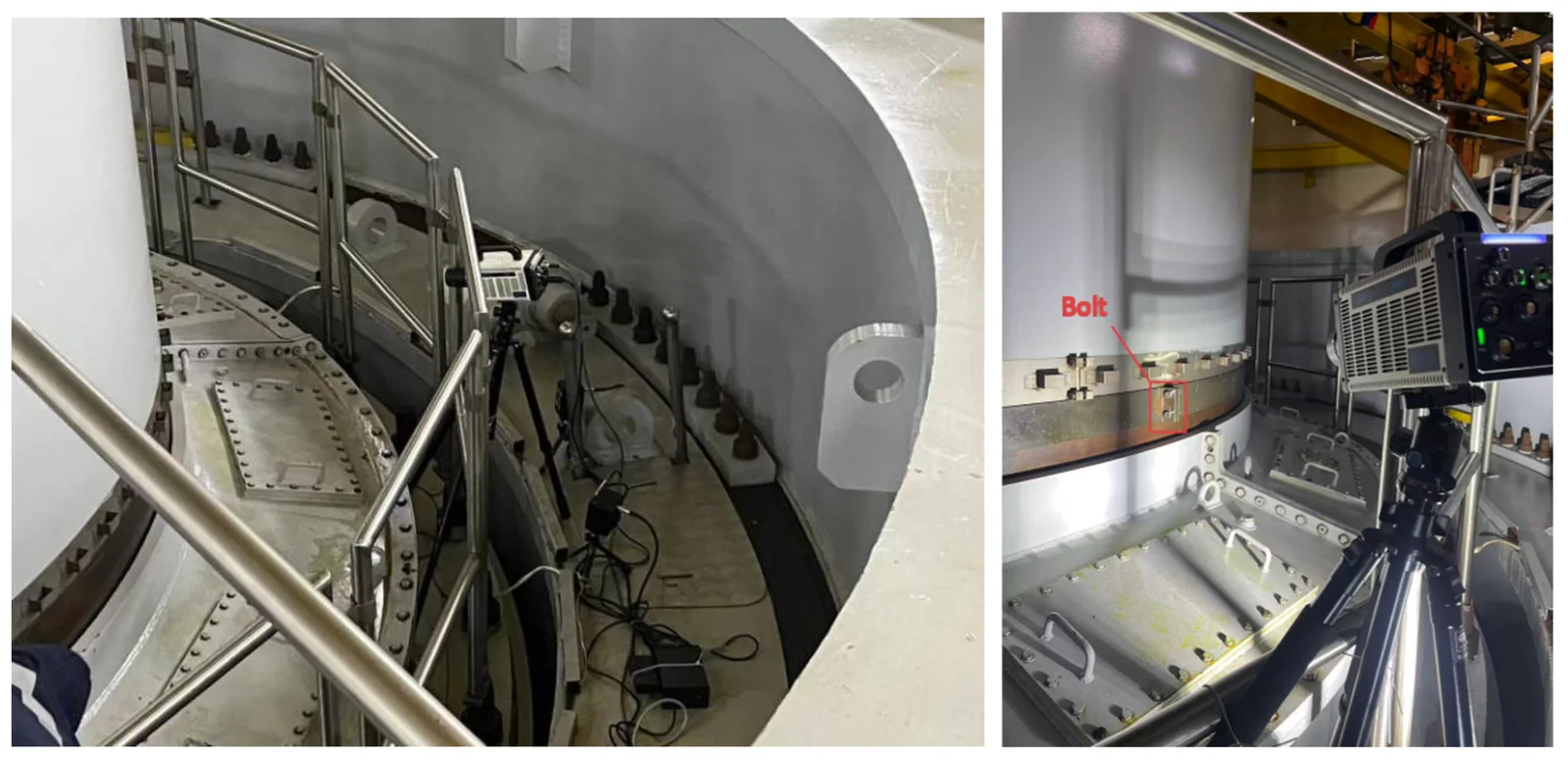
Field camera and illumination arrangement.

Figure 18 shows the field acquisition arrangement. A high-speed camera was mounted on a bracket secured by three magnetic anchors and tethered to the safety fence. Two 120 W LED lamps provided supplementary illumination, and rotor-clamp bolts were recorded from the available viewpoints.

**Figure 19:**
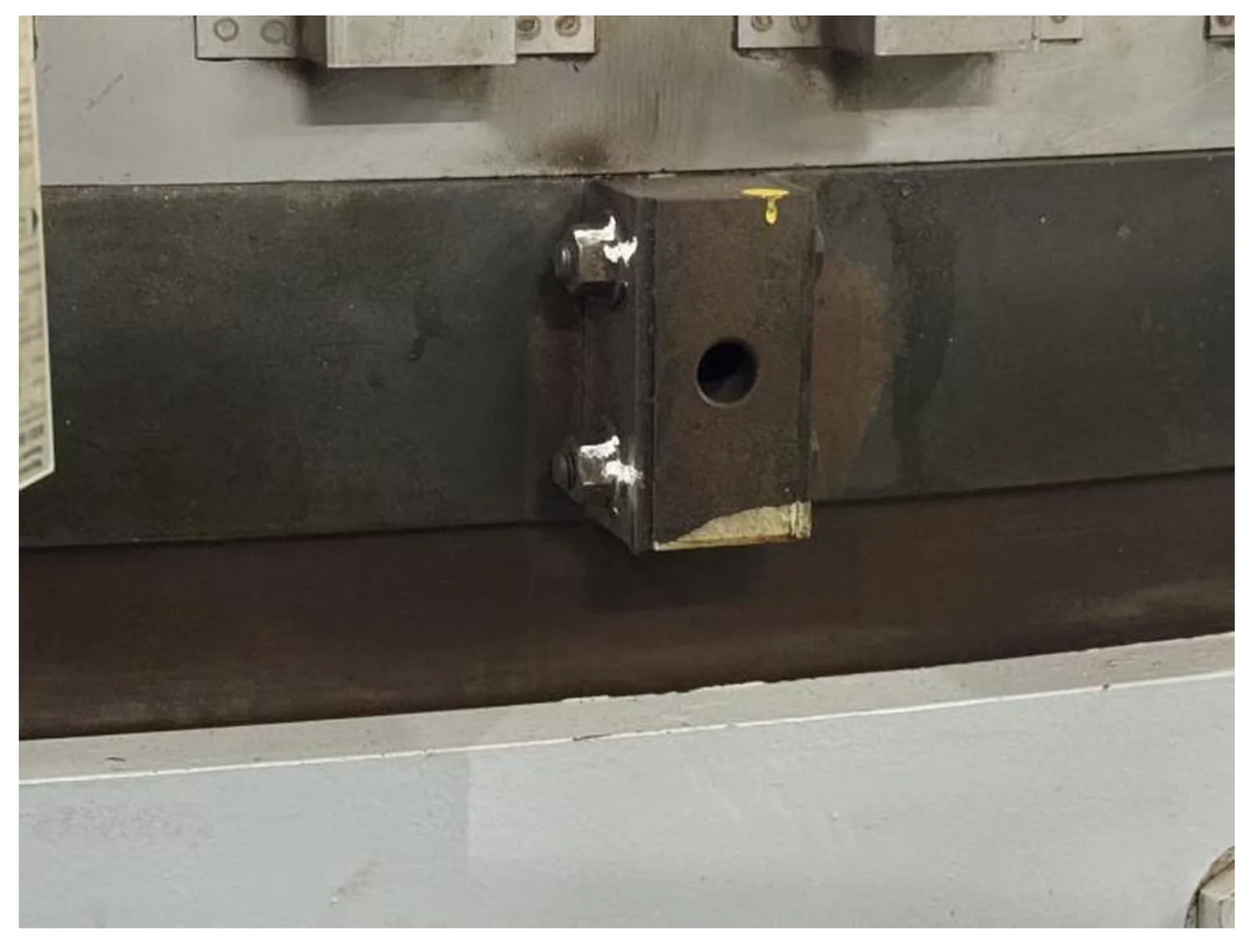
Representative M12 field bolts.

The field environment differed substantially from the laboratory setup. Illumination was strongly non-uniform, metallic reflection and shadows were pronounced, and camera placement produced oblique views. The M12 rotor-clamp bolts were also considerably smaller than the laboratory M42 specimens. A total of 850 representative field frames were selected from the recorded videos (Figure 19).

**Figure 20:**
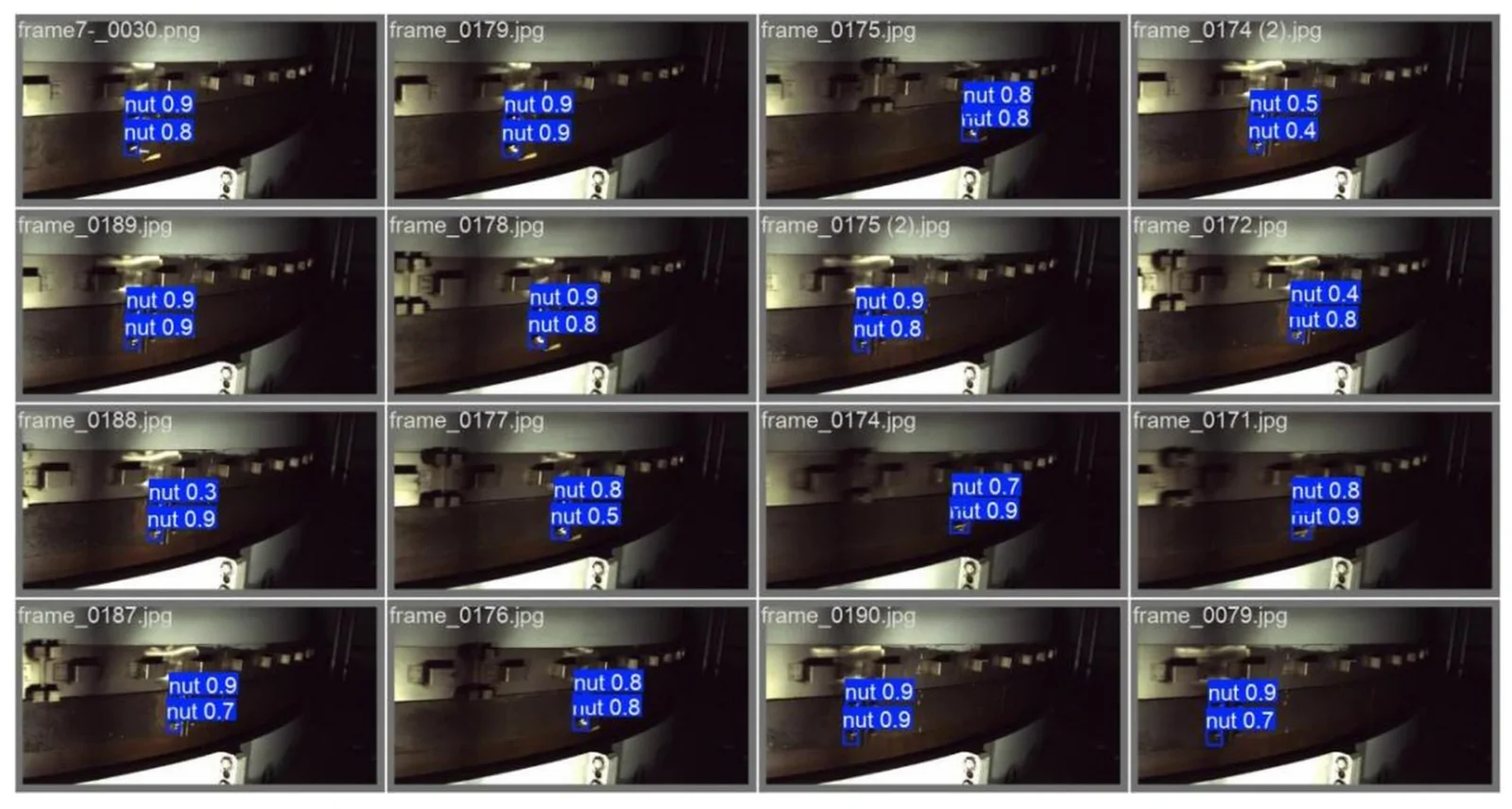
Representative YOLO-SE field detections.

YOLO-SE first localized bolts in the field images, after which the three segmentation models processed the cropped ROIs. On the field validation dataset, YOLO-SE achieved 97.88% precision, 98.21% recall, and 99.41% mAP50. Bolt localization succeeded in more than 99% of the 850 field images (Figure 20).

The on-site ROIs were used to train and validate site-specific segmentation models. As shown in Table 12, mIoU exceeded 92% for all three targets. TL-IoU was 90.16% for the nut region, 84.76% for the nut-side marking line, and 87.47% for the base-side marking line. The lower line-level scores coincide with the more complex backgrounds, oblique views, and reduced field contrast.

**Figure 21:**
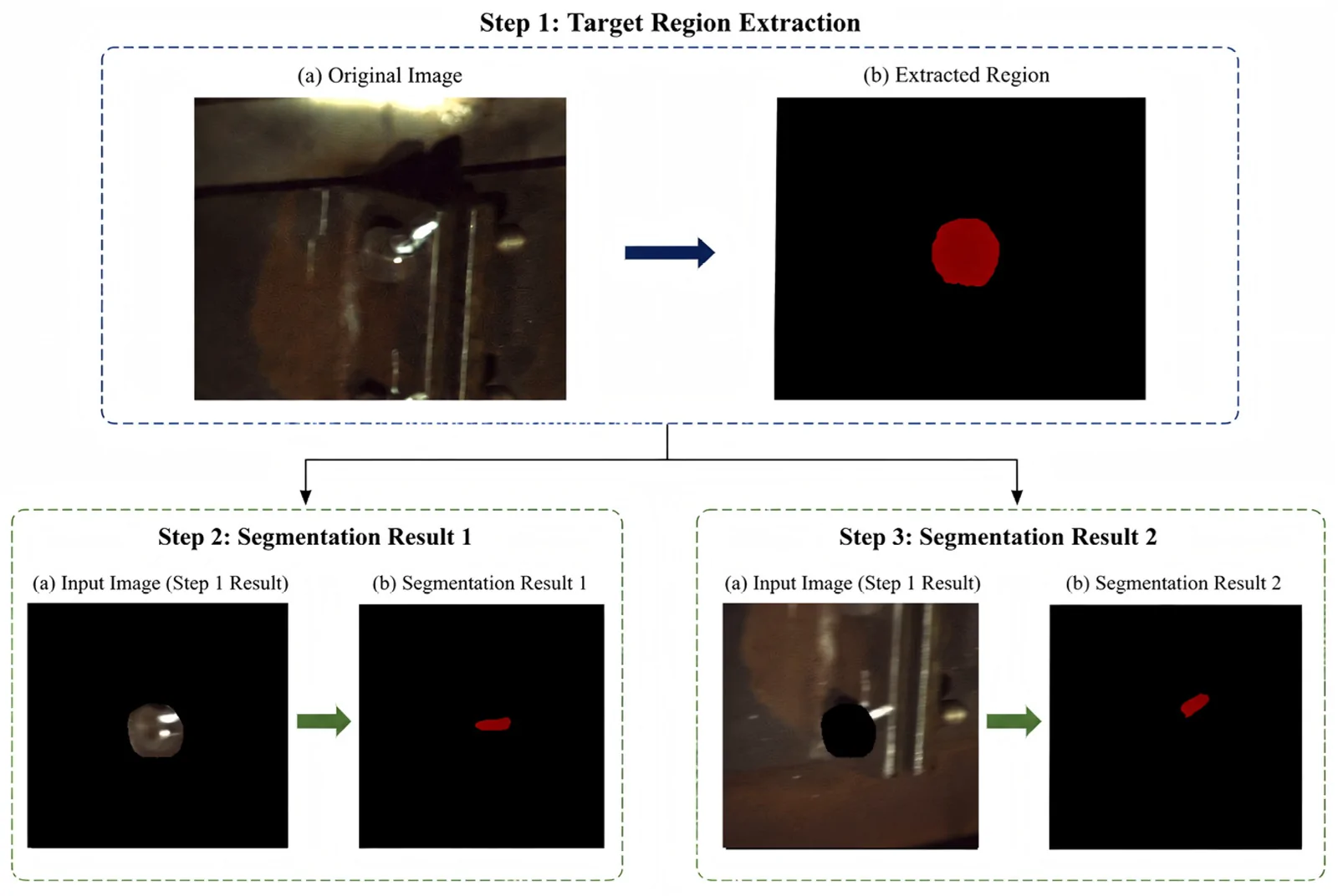
Three-stage segmentation of representative field data.

Figure 21 presents representative field masks. The three-stage segmenter delineated the nut region and both marking-line segments despite the small target size, complex backgrounds, and locally weak boundaries.

Because the field models were trained and validated on on-site data, this experiment assesses site-specific applicability rather than zero-shot laboratory-to-field transfer. The field images differed from the laboratory data in bolt specification (M12 versus M42), imaging distance, viewpoint, illumination, background, and contamination. YOLO-SE retained 99.41% field mAP50, whereas marking-line TL-IoU decreased to 84.76–87.47% from 95.38% for the laboratory complete segmenter. Micro-level boundary reconstruction was therefore more sensitive than macro-level localization to site-specific image formation.

Qualitative inspection of lower-performing field cases showed boundary fragmentation and local under-segmentation when oblique viewing; oil contamination, shadow, or metallic reflection reduced line contrast. Because these cases were not collected as a prospectively balanced failure set, they are reported as descriptive failure modes rather than as a controlled robustness experiment.

### 3.5. Discussion

The results support task-conditioned decomposition as the principal design contribution. Full-frame bolt localization and ROI-level marking-line segmentation are limited by different image-formation and representation problems. At panoramic scale, performance depends on retaining sparse bolt evidence while suppressing reflection-dominated background responses. Within normalized ROIs, the dominant challenge shifts to reconstructing discontinuous foreground boundaries under severe class imbalance. Separating these objectives reduces the need for a single network to resolve both scale regimes simultaneously.

Ablation results clarify the complementary roles of the proposed components. Independent detector ablations showed that SPD-Conv and EMA improved different aspects of precision and recall. The factorial segmentation analysis identified Dice cross-entropy loss as the largest independent contributor to TL-IoU, whereas A-ASPP primarily improved mIoU and slightly reduced TL-IoU when added to Model A. Region-level overlap and slender-line recovery should therefore be treated as related but distinct objectives. These accuracy gains also introduced computational costs. YOLO-SE increased parameter count by 73.1% and FLOPs by 40.7%. The complete cascade achieved 31.4 FPS on the reported GPU but was not in real time on the CPU.

Controlled robustness and field results indicate that macro-level localization is less sensitive than micro-level boundary reconstruction to changes in image formation. YOLO-SE detected 3923 of 3927 controlled samples and retained 99.41% mAP50 in the field dataset. By contrast, field marking-line TL-IoU decreased to 84.76–87.47%. Because the field segmenters were trained on on-site data, these results demonstrate site-specific applicability rather than zero-shot transfer. Validation on additional turbine units is required before broader generalization can be claimed.

Motion blur remains the most significant unverified stressor. Unlike variations in distance or illumination conditions, the directional integration process during exposure may eliminate the contrast and boundary details of marker lines before any inference can be made. In this experiment, data acquisition was not independently adjusted for rotor speed, exposure time, blur direction, or blur length due to constraints imposed by field conditions; furthermore, our high-speed camera configuration ensures full coverage of both frame rates and exposure times across the entire rotor speed range, guaranteeing the acquisition of relatively clear images. Consequently, occasional blurred frames cannot provide analysis results based on severity metrics. Therefore, we do not claim that this study demonstrates robustness against severe motion blur. To conduct a precise evaluation, it is necessary to jointly control the aforementioned acquisition variables and compare the actual captured blurred images with matched synthetic enhanced images. These aspects represent promising directions for future research.

Several deployment questions also remain outside the present evidence. Occlusion, sensor noise, and image compression were not independently controlled, and external validation was limited to one hydropower site. Embedded-device power consumption and continuous-operation stability were not measured. These limitations define the next validation stages: multi-site acquisition, calibrated degradation tests, domain adaptation, and hardware-specific assessment of latency, memory, power, and long-term stability.

## 4. Conclusions

This study presents a task-decoupled vision framework that localizes hydropower-rotor bolts at panoramic scale and reconstructs anti-loosening marking lines within scale-normalized ROIs. Its main contribution is the alignment of detector and segmenter design choices with the distinct degradation mechanisms of the two spatial scales.

In laboratory experiments, YOLO-SE achieved 99.4% mAP50 and 99.8% recall, while the complete segmenter achieved 98.67% mIoU and 95.38% TL-IoU. Site-specific validation on M12 rotor-clamp bolts retained 99.41% detection mAP50, although marking-line TL-IoU decreased to 84.76–87.47% under field imaging conditions.

Together, these results support the framework as a feature-extraction stage for subsequent loosening-angle measurement under the evaluated laboratory and single-site field conditions. They do not establish zero-shot transfer to other hydropower sites or unrelated industrial tasks.

The principal unresolved limitation is motion blur, which was observed but not independently controlled during physical acquisition. Severe directional smear may remove marking-line evidence before inference, so the reported robustness to distance, illumination, height, and viewpoint should not be interpreted as blur robustness. Controlled occlusion, sensor noise, image compression, embedded power consumption, and long-term operational stability also remain untested.

Future work will prioritize a calibrated motion-blur protocol that varies rotor speed, exposure time, smear direction, blur length, and marking-line contrast. Detection recall, TL-IoU, and BF1 should be reported as functions of blur severity and compared between physical and synthetic blur. Subsequent validation will extend to multiple sites, embedded hardware, domain adaptation, power consumption, and continuous operation.

## Figures and Tables

**Table 1:** Comparison of representative vision-based bolt-loosening studies and the present framework.

Study	Primary Task	Core Approach	Strength	Limitation Relative to This Study
Cha et al. [4]	Loosening classification	Hough transform + SVM	Interpretable handcrafted geometry	Sensitive to illumination and weak edges
Sun et al. [7]	Angle estimation	YOLOv5 + visual markers	High-accuracy detection under varied imaging	Requires dedicated circular markers
Li et al. [10]	Angle estimation	Semantic segmentation + perspective correction	Arrangement-independent geometric measurement	Depends on sufficiently complete local contours
Lei et al. [11]	Real-time angle estimation	YOLOv10-S + Fast-SCNN	Real-time two-stage pipeline	Limited analysis of severe feature degradation
Wu et al. [12]	Keypoint-based measurement	High-resolution cross-scale Transformer	Global context and fine-detail modeling	Targets bolt keypoints rather than marking-line masks
Meng et al. [13]	Turbine-rotor monitoring	YOLOv8 + DeepSORT + DeepLabV3+	Field validation on a hydropower rotor	Focuses on detection, tracking, and angle calculation
Lin et al. [14]	Explainable detection	Visual target + BoltYOLO + Grad-CAM	Improves interpretability	Does not address fine-line semantic reconstruction
Present study	Degraded feature extraction	YOLO-SE + improved DeepLabV3+	Couples small-target localization with high-fidelity line segmentation	Evaluation remains bounded to laboratory and single-site field data

**Table 2:** Analog experimental data acquisition checklist.

Variable	Level	Groups	Images	Percentage
Shooting Distance (d, cm)	d = 20	20	600	10.5%
	d = 30	35	1050	18.4%
	d = 40	45	1350	23.8%
	d = 50	55	1650	28.9%
	d = 60	20	600	10.5%
	d = 70	15	450	7.9%
	Distance total	190 groups	5700 images	100%
Light Intensity (I,%)	I = 0%	38	1140	20%
	I = 10%	38	1140	20%
	I = 20%	38	1140	20%
	I = 30%	38	1140	20%
	I = 40%	38	1140	20%
	Illumination total	190 groups	5700 images	100%
Camera Height (h, cm)	h = 10	15	450	7.9%
	h = 20	20	600	10.5%
	h = 30	25	750	13.2%
	h = 40	45	1350	23.7%
	h = 50	50	1500	26.3%
	h = 60	35	1050	18.4%
	Height total	190 groups	5700 images	100%
Top View Angle (θ, °)	θ = 10°	35	1050	18.4%
	θ = 20°	55	1650	28.9%
	θ = 30°	55	1650	28.9%
	θ = 40°	35	1050	18.4%
	θ = 50°	10	300	5.4%
	Angle total	190 groups	5700 images	100%
Total Sample Size	Combined conditions	190 groups	5700 images	—

**Table 3:** Training hardware and software environment.

Environment Item	Configuration
CPU	Intel Core i9-13900HX
GPU	NVIDIA RTX 4090
System Memory	32 GB DDR5
Operating System	Windows 11
Python Version	Python 3.11
CUDA Version	CUDA 12.4
PyTorch Version	PyTorch 2.1

**Table 4:** Stepwise ablation results for YOLO-SE.

Experiment	YOLO26n	SPD-Conv	EMA	Precision (%)	Recall (%)	mAP50 (%)
1	✓			94.2	96.4	96.2
2	✓	✓		96.8	98.7	99.1
3	✓		✓	94.4	97.8	99.2
4	✓	✓	✓	97.7	99.8	99.4

**Table 5:** Comparison with representative object-detection algorithms.

Model	Precision (%)	Recall (%)	mAP50 (%)	Parameters (M)
Faster R-CNN	88.4	86.1	87.5	41.8
YOLOv8n	94.1	95.5	97.3	6.1
YOLO11n	94.5	94.4	95.8	5.3
RT-DETR	94.6	94.1	95.1	64.7
YOLO-SE (ours)	97.7	99.8	99.4	9.0

**Table 6:** Primary segmentation ablation results.

Model	Backbone	Module Additions	Loss Function	mIoU (%)	TL-IoU (%)
Baseline	ResNet-50	Native ASPP	CE Loss	96.65	93.36
Model A	MobileViT2	Native ASPP	DiceCELoss	97.69	95.42
Ours	MobileViT2	A-ASPP	DiceCELoss	98.67	95.38

**Table 7:** Computational cost and inference efficiency under a unified profiling protocol.

Model/Stage	Input	Parameters (M)	FLOPs (G)	Peak GPU Memory (MB)	GPU Latency (ms)	CPU Latency (ms)	FPS
YOLO26n	2048 × 1024	2.6	31.2	892	3.54	62.8	282.5
YOLO-SE	2048 × 1024	4.5	43.9	1108	4.27	78.6	234.2
DeepLabV3+ baseline	640 × 640	39.6	181.4	2136	13.84	286.0	72.3
Model A	640 × 640	15.7	52.8	1354	8.42	129.6	118.8
Improved DeepLabV3+	640 × 640	16.2	54.6	1468	9.16	137.9	109.2
Complete cascade	Full frame + three ROIs	53.1	207.7	2380	31.8	492.3	31.4

**Table 8:** Expanded independent ablation of the segmentation components.

Configuration	MobileViT2	Dice CE	A-ASPP	mIoU (%)	TL-IoU (%)	Parameters (M)	FLOPs (G)
Baseline				96.65	93.36	39.6	181.4
Backbone only	✓			97.10	94.20	15.7	52.8
Loss only		✓		97.34	94.83	39.6	181.4
A-ASPP only			✓	97.26	93.74	40.1	183.2
Model A	✓	✓		97.69	95.42	15.7	52.8
Backbone + A-ASPP	✓		✓	98.02	94.48	16.2	54.6
Loss + A-ASPP		✓	✓	98.31	95.06	40.1	183.2
Complete model	✓	✓	✓	98.67	95.38	16.2	54.6

**Table 9:** Statistical stability of the principal detector and segmentation comparisons.

Model	Primary Metric	Current Single-Run Value	Runs (n)	Mean ± SD	95% CI	Effect vs. Baseline	*p*-Value
YOLO26n	mAP50 (%)	96.2	5	96.12 ± 0.24	[95.82, 96.42]	Reference	–
YOLO-SE	mAP50 (%)	99.4	5	99.32 ± 0.10	[99.20, 99.44]	+3.20 pp	<0.001
DeepLabV3+	mIoU (%)	96.65	5	96.60 ± 0.18	[96.38, 96.82]	Reference	–
Complete segmenter	mIoU (%)	98.67	5	98.61 ± 0.09	[98.50, 98.72]	+2.01 pp	<0.001
DeepLabV3+	TL-IoU (%)	93.36	5	93.29 ± 0.31	[92.91, 93.68]	Reference	–
Complete segmenter	TL-IoU (%)	95.38	5	95.34 ± 0.22	[95.07, 95.61]	+2.05 pp	<0.001

**Table 10:** Detection-rate robustness test.

Test Samples	Variable Scope	Missed Detections	Detection Rate
3927 frames	Distance, height, viewpoint, and illumination	4 frames	99.898%

**Table 11:** Segmentation robustness-test results.

Segmentation Target	Test ROIs	Physical Variables	mIoU	Boundary F1
Nut region	5573	Angle, Height, Distance, Lighting	0.995	0.998
Nut-side marking line			0.973	0.952
Base-side marking line			0.976	0.989

**Table 12:** Field-data segmentation performance.

Target	mIoU	TL-IoU
Nut region	94.84%	90.16%
Nut-side marking line	92.34%	84.76%
Base-side marking line	93.71%	87.47%

## Data Availability

The laboratory and field bolt-image datasets are not publicly posted because the field component contains site-specific industrial imagery and manufacturing information. De-identified laboratory images, non-sensitive cropped field ROIs, trained-model configurations, and evaluation code may be requested for non-commercial research from the corresponding authors (ran_yingbing@cypc.com.cn, guo.river@whu.edu.cn, or coderyuan@whu.edu.cn). Access is subject to institutional review of site confidentiality constraints and, where required, execution of a data use agreement that prohibits re-identification or redistribution.
